# Synthesis of PEG‐Polycycloether Block Copolymers: Poloxamer Mimics Containing a Rigid Helical Block

**DOI:** 10.1002/advs.202310277

**Published:** 2024-03-23

**Authors:** Jean‐Baptiste Masclef, Joëlle Prunet, Bernhard V. K. J. Schmidt

**Affiliations:** ^1^ School of Chemistry, Joseph Black Building University of Glasgow Glasgow G12 8QQ UK

**Keywords:** emulsion, helicity, poloxamer mimic, polycycloether, self‐assembly

## Abstract

Poloxamers are amphiphilic block copolymers consisting of poly(ethylene glycol) (PEG) and poly(propylene glycol) segments. Their self‐assembly and interfacial properties are tied to the relative hydrophilicity and hydrophobicity of each block and can therefore be adjusted by changing block lengths. Here, a series of PEG‐polycycloether block copolymers is synthesized that have the same structure as a poloxamer, but they encompass a rigid polycyclic backbone as the hydrophobic block. A variety of polymer structures are synthesized, for example diblock or triblock architectures, with/without olefinic units, atactic or isotactic backbone, and different block lengths. Due to their amphiphilicity, self‐assembly into spherical aggregates (diameters ranging from 64 to 132 nm) at low concentrations (critical aggregation concentration as low as 0.04 mg mL^−1^) is observed in water. Low surface tensions (as low as 26.7 mN m^−1^) are observed as well as the formation of stable high internal phase emulsions (HIPEs) irrespective of the oil/water ratio. This contrasts with the properties of the commonly used poloxamers P188 or P407 and illustrates the significance of the rigid polycycloether block. These new colloidal properties offer new prospects for applications in emulsion formulations for biomedicine, cosmetics, and the food industry.

## Graphical abstract

The synthesis of poloxamer‐mimicking block copolymers that include a rigid polycycloether block gives access to new colloidal properties. High‐internal phase emulsions are obtained in the form of oil‐in‐water emulsions. These new colloidal properties open‐up opportunities for applications in emulsion formulations for biomedicine, cosmetics, and the food industry.

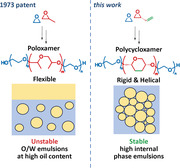

## 1 Introduction

Poloxamers were patented in 1973 by Irving Schmolka at BASF.^[^ ^1^ ^]^ A poloxamer can be described as a triblock copolymer, composed of one block of poly(propylene glycol) (PPG) sandwiched between two blocks of poly(ethylene glycol) (PEG). These materials were originally developed to form aqueous gels with potential uses in biomedical or cosmetic applications.^[^ ^2^, ^3^, ^4^, ^5^, ^6^ ^]^ Since then, the use of poloxamers has increased dramatically, reaching a total market volume of $225 million in 2021.^[^ ^7^ ^]^ One major feature of poloxamers is their surface‐active properties. Their amphiphilicity, ease of production, and biocompatibility led to their extensive use in the cosmetics industry as emulsifiers.^[^ ^8^, ^9^, ^10^, ^11^ ^]^ In recent years, poloxamers have also been used in biomedical applications such as drug delivery, gene therapy, or even bioprocess applications, to protect cell cultures from stressful shear conditions.^[^ ^12^, ^13^, ^14^, ^15^, ^16^ ^]^

Previously, to tailor poloxamer properties to a specific application, poloxamers were synthesized with various PEG and PPG chain lengths, effectively modifying the amphiphilicity or hydrophilic‐lipophilic balance (HLB) of the block copolymer. Poloxamers are generally designed by the letter “P” followed by three digits, the first two digits multiplied by 100 gives the molecular weight of the hydrophobic middle block, while the last digit multiplied by 10 denotes the % weight content of the hydrophilic PEG block. For example, P407 is a commonly used poloxamer with a 4000 g mol^−1^ PPG block and a 70% PEG content.

In order to produce poloxamers with additional properties, poloxamers can be functionalized with various end‐groups.^[^ ^17^, ^18^ ^]^ For example, Lu and coworkers reported the substitution of the hydroxy end‐group of a P407 poloxamer with an amino group.^[^ ^19^ ^]^ The amino end‐group improved the mucoadhesive properties of the polymer and its subsequent use as an in situ hydrogel drug delivery system for vaginal administration. Katas and coworkers used P407 end‐groups to attach a folic acid‐targeting ligand to produce a polymeric drug delivery system, which could target cancer cells due to their over‐expression of folic acid receptors on the cell membrane.^[^ ^20^ ^]^ The functionalization of the poloxamer led to improved cellular uptake and minimal toxicity of the micelles and their cargo. Poloxamers can also be functionalized by adding a reactive linker between each block. For example, Frey and coworkers described the synthesis of a cleavable PEG‐*b*‐PPG‐*b*‐PEG copolymer containing two pH‐responsive acetal linkers in the polymer backbone.^[^ ^21^ ^]^ The addition of the acetal linker did not prevent the triblock copolymer from showing good surface‐active behaviors. The polymer was used as a surfactant for the miniemulsion polymerization of styrene, triggering precipitation via a change of pH. Additionally, replacing the PPG block with poly(butylene glycol) has been found advantageous in cases where a more hydrophobic block was needed,^[^ ^22^, ^23^ ^]^ granting a lower critical micellar concentration (CMC) compared to the corresponding PPG‐based block copolymers. In particular, Green and coworkers have developed poly(butylene glycol)‐*b*‐PEG to form high‐internal phase emulsions (HIPE)s and polymerized HIPE foams.^[^ ^24^ ^]^ The block copolymer was able to stabilize an HIPE at a concentration as low as 0.006 wt%. HIPEs are systems in which the dispersed phase has a fraction volume higher than 0.74. This high volume can lead to a morphology change from spherical droplets to polyhedrons.^[^ ^25^ ^]^ In addition, HIPEs have unusual properties, like high viscosity and viscoelastic rheological behaviors.^[^ ^26^, ^27^ ^]^ Frequently, HIPEs are used as a polymerization template to control the microstructure of a material.^[^ ^28^, ^29^, ^30^, ^31^ ^]^ Currently, new poloxamer properties have been obtained by either tuning their hydrophilicity/hydrophobicity ratio (by tuning chain lengths or introducing more hydrophobic blocks) or by adding functional groups.

**Figure 1:**
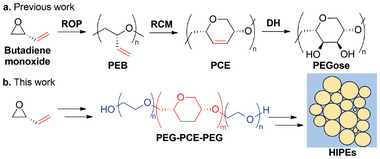
a) Synthesis Scheme of atactic PEGose previously performed in the Prunet group via ring‐opening polymerization (ROP) of butadiene monoxide into poly(epoxybutene) (PEB), ring‐closing metathesis (RCM) into polycycloether (PCE) then dihydroxylation (DH) into PEGose and b) simplified Scheme of the work achieved here: the synthesis of PEG‐PCE block copolymers and the formation of high‐internal phase emulsions (HIPEs).

The synthesis of our poloxamer mimics was inspired by the Prunet and Shaver groups’ work on polycycloethers and PEGose (See **Figure** **1**a).^[^ ^32^ ^]^ They recently described a way to obtain a polycycloether (PCE) by using ring‐closing metathesis as a post‐polymerization functionalization tool. Ring‐opening polymerization (ROP) of butadiene monoxide followed by ring‐closing metathesis (RCM) of the resulting poly(epoxybutene) (PEB) furnished a polycycloether containing an internal alkene that was functionalized via dihydroxylation (DH). The resulting polymer, coined PEGose, was shown to have a helical conformation when isotactic.

In this work, the methodology to produce the PCEs was leveraged in order to obtain a poloxamer mimic, consisting of either diblock or triblock copolymers with PEG blocks and a saturated polycycloether block (SPCE), with no additional functional group. The diblock copolymers containing an unsaturated PCE block are less similar to poloxamers than the corresponding triblock copolymers containing an SPCE block. Nevertheless, these polymers exhibit an identical HLB value to that of the corresponding poloxamers, but a different rigidity. The increase in rigidity moving from PPG/PEG to PCEs can be deduced from persistence lengths, for example PEG has a persistence length of 0.38 nm,^[^ ^33^ ^]^ rigid polysaccharides like iota‐carrageenan and xanthan gum have a high persistence length of 23–26 nm as well as more flexible polysaccharides like locust bean gum and guar gum with random coil conformations still have a persistence length of 3 nm.^[^ ^34^ ^]^ PCEs with helical or random coil conformations come close to polysaccharides, which leads to increased persistence lengths and higher rigidity than PEG or PPG. The introduction of a rigid block could potentially induce new self‐assembly behaviors and interfacial properties, and good emulsification properties such as high internal phase (See Figure 1b).

## 2 Results and Discussion

### 2.1 Synthesis of the Block Copolymers

**Figure 2:**
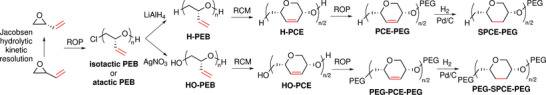
Synthesis scheme of PEG‐*b*‐PCEs.

**Figure 3:**
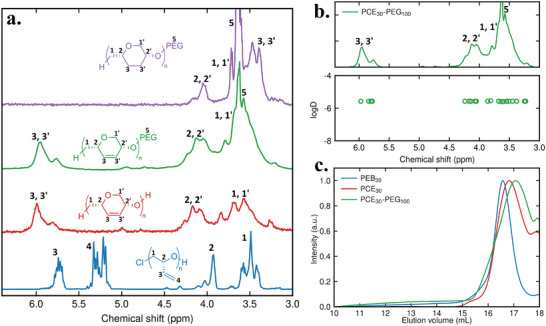
a) ^1^H NMR of the synthesized polymers PEB_30_, PCE_30_, PEG_100_‐*b*‐PCE_30_ and PEG_100_‐*b*‐SPCE_30_ in CDCl_3_, b) DOSY ^1^H NMR of the block copolymer PEG_100_‐*b*‐PCE_30_ in CDCl_3_, c) Size‐exclusion chromatography traces of PEB_30_, PCE_30_ and PEG_100_‐*b*‐PCE_30_ measured in THF.

The atactic block copolymers were synthesized from racemic butadiene monoxide (See **Figure** **2**). The synthesis of the isotactic block copolymers required enantiomerically pure butadiene monoxide as a monomer. The *R* enantiomer of this epoxide (shown in Figure 2) was obtained by Jacobsen hydrolytic kinetic resolution of racemic butadiene monoxide with a chiral cobalt‐salen catalyst, which converted the undesired *S* enantiomer to the corresponding diol.^[^ ^35^ ^]^ ROP was then performed on racemic and (*R*)‐butadiene monoxide to give atactic and isotactic PEB, respectively. The initiator and catalyst for the polymerization, tetraphenylporphyrin aluminum chloride (TPPAlCl), was synthesized from tetraphenylporphyrin (TPPH_2_)^[^ ^36^ ^]^ by mixing with 1 M diethylaluminum chloride solution. Racemic or enantiopure butadiene monoxide was added and the mixture was stirred at ambient temperature to afford PEB, which was purified by preparative size‐exclusion chromatography (SEC) using a methanol/dichloromethane mixture. A 1:30 initiator/monomer ratio was used to obtain PEB_30_. The number of repeating units (30 repeating units, 2100 Da) was chosen to mimic the hydrophobic block in P188 (31 propylene glycol repeating units, 1800 Da), one of the commercially most used poloxamers. The polymer molecular weight was estimated by SEC against poly(styrene) calibration and ^1^H nuclear magnetic resonance (NMR) through end‐group peaks integration (See **Table** **1**). Furthermore, the polymer structure was confirmed by ^1^H NMR (See **Figure** **3**a; Figure S3, Supporting Information).

**Table 1:** Theoretical polymer molecular weights and experimental polymer molecular weights are measured by SEC and ^1^H NMR. Critical Aggregation Concentrations (CACs) are measured by fluorescence spectroscopy and surface tensions are measured by droplet shape analysis (See Figures S6 and S7, Supporting Information).

Polymer	*M_n_* Theo^a)^ [kDa]	*M_n_* SEC^b)^ [kDa]	*M_n_* NMR^c)^ [kDa]	Đ^b)^	CAC^d)^ [mg/mL]	Surface tension^e)^ [mN/m]
**aPEB_30_**	2.1	2.4	2.7	1.19	–	–
**iPEB_30_**	2.1	2.3	2.6	1.18	–	–
**aPCE_30_**	1.7	2.1	–	1.21	–	–
**iPCE_30_**	1.7	2.0	–	1.22	–	–
**aPCE_30_‐PEG_100_**	6.1	1.9	6.7	1.14	0.12	32.5
**aPCE_30_‐PEG_200_**	10.5	2.6	9.6	1.39	0.15	36.6
**iPCE_30_‐PEG_100_**	6.1	2.9	7.5	1.18	0.04	30.8
**iPCE_30_‐PEG_200_**	10.5	2.0	13.2	1.31	0.22	37.7
**aSPCE_30_‐PEG_100_**	6.1	2.9	6.7	1.32	0.05	26.7
**iSPCE_30_‐PEG_100_**	6.1	2.3	7.5	1.14	0.21	35.1
**PEG_50_‐aPCE_30_‐PEG_50_**	6.1	2.0	6.2	1.18	0.15	42.0
**PEG_50_‐aSPCE_30_‐PEG_50_**	6.1	2.5	9.2	1.37	0.04	42.7
**PEG_50_‐iPCE_30_‐PEG_50_**	6.1	2.0	5.1	1.27	0.11	46.5

^a)^ calculated from starting material equivalents;

^b)^ measured via SEC in THF with a 1 mL min^−1^ flow rate;

^c)^ measured via ^1^H NMR in CDCl_3_;

^d)^ measured via fluorescence spectroscopy using pyrene as a fluorescence probe;

^e)^ measured at 20 °C using a contact angle goniometer and droplet shape analysis.

**Figure 4:**
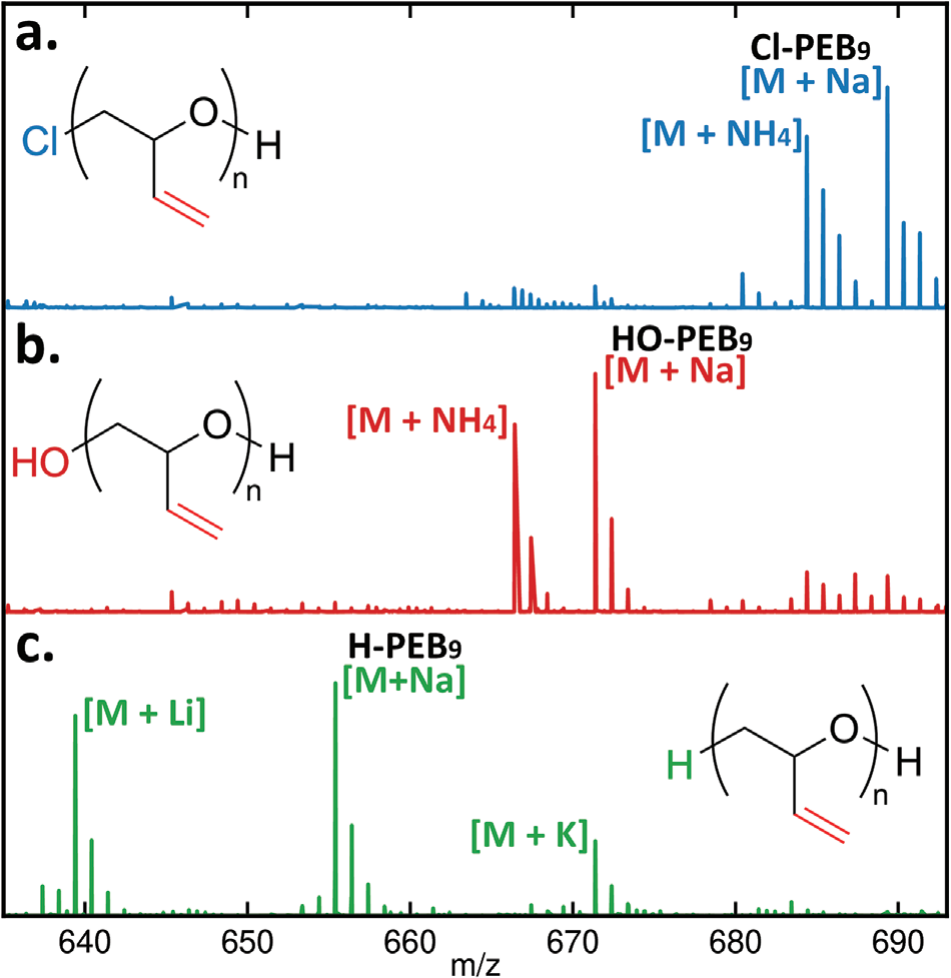
ESI‐MS spectra of poly(epoxybutene) with a) chloride end‐group, b) hydroxy end‐group, and c) hydrogen end‐group.

A PEB with only 15 repeating units (1.0 kDa) was also synthesized to allow for electrospray‐ionization mass spectrometry (ESI‐MS) characterization (See **Figure** **4**; Figure S1 and Table S1, Supporting Information). ESI‐MS confirmed the structure of PEB repeating units and its chloride and hydroxy end‐groups (See Figure 4a). Following the ROP of butadiene monoxide, H‐PEB or HO‐PEB were obtained by modification of the PEB chloride end‐group. In order to obtain a triblock copolymer, HO‐PEB was synthesized to be used later as a macroinitiator for the ROP of ethylene oxide. While Cl‐PEB could have been used, H‐PEB was synthesized to prevent any S_N_2 side‐reaction during the ROP of ethylene oxide, and also because it possesses a similar end‐group as those commonly found in PPG‐based block copolymers. H‐PEB was obtained through the reduction of the chloride end‐group with 10 equivalents of lithium aluminum hydride (LiAlH_4_) in THF. The reaction conversion could not be determined by ^1^H NMR but ESI‐MS confirmed both the presence of H‐PEB and the absence of any remaining Cl‐PEB (See Figure 4c). Sodium, lithium, and potassium adducts from the use of LiAlH_4_ and the Rochelle salt solution could be observed in the mass spectrum. HO‐PEB was obtained using silver nitrate in a water/ethanol mixture. Similarly, analysis by ^1^H NMR did not indicate if the reaction had proceeded but ESI‐MS confirmed the conversion of the starting polymer and the formation of the HO‐PEB structure (See Figure 4b). Small peaks corresponding to Cl‐PEB were still visible on the HO‐PEB mass spectrum which might lead to some diblock copolymer impurities after the ring‐opening polymerization of ethylene oxide. SEC showed that both H‐PEB and HO‐PEB have a similar molecular weight after the end‐group modification, thus confirming the absence of polymer degradation.

The ring‐closing metathesis of each PEB polymer was performed in dichloroethane, using the Grubbs II catalyst with a 5 mol% catalyst loading, under reflux. Dichloroethane, as opposed to dichloromethane, was chosen as the solvent due to its higher boiling point. The polymer concentration was kept at 0.2 mM (calculated per olefin) as higher concentrations led to cross‐metathesis between different polymer chains. Reaction completion was confirmed by ^1^H NMR after 5 days. The conversion could be calculated by the relative integration of internal and external alkene peaks. The polymers were purified by preparative SEC and characterized by ^1^H NMR as well as SEC. SEC showed a decrease in polymer molecular weight, confirming the loss of an ethylene molecule for every two vinyl groups (See Figure 3c and Table 1; Figure S2, Supporting Information). The isotactic polymers encompass *cis* 1,4‐disubsituted six‐membered rings, which induce helicity. Circular dichroism (CD) confirmed the helicity of these isotactic polymers (See Figure S5, Supporting Information). It should be noted that the number of repeating units halves after RCM from PEB to PCE. To be consistent we kept the subscript of PCE the same as for the PEB block. The synthesis of the PEG block was achieved through ROP of ethylene oxide using the previously synthesized PCE as a macroinitiator and sodium hydride as a base (heated at 50 °C over 2 days). A diblock copolymer was obtained when H‐PCE was used as the macroinitiator for the ROP of ethylene oxide and a triblock copolymer was formed with HO‐PCE, due to the protic hydroxy end‐group on both ends. The block copolymer, now water‐dispersible, was purified by dialysis against water. In the ^1^H NMR spectrum, the peak at 3.6 ppm confirmed the presence of the PEG block (See Figure 3a).

Diffusion‐ordered spectroscopy (DOSY) indicated that the diffusion coefficient associated with the PEG peak was the same as diffusion coefficients associated with PCE peaks, thus confirming the presence of a block copolymer structure, with the absence of PEG homopolymers (See Figure 3b; Figure S4, Supporting Information). SEC did not indicate an increase of the molecular weights, contrary to predicted, but a shift toward lower elution times has been previously reported in cases of block copolymer formation.^[^ ^37^ ^]^ In the current case, the measured molecular weight was even slightly lower than before the ROP. These results could be explained by various reasons. First, the column is calibrated with poly(styrene) standard, the structure of which differs greatly from the analyzed amphiphilic block copolymer. Second, block copolymer architectures have been reported to appear with a lower molecular weight than expected due to conformational effects arising from two blocks of different solubilities leading to a smaller hydrodynamic radius than the starting block.^[^ ^37^ ^]^ In any case, the presence of a monomodal SEC peak, combined with the correct ^1^H NMR spectrum and a single diffusion coefficient from all DOSY polymer peaks confirm the presence of the desired structure (See Figure 3). As previously mentioned, the molecular weight of the PCE homopolymer was estimated by SEC and ^1^H NMR. The block copolymer molecular weight was then estimated by comparing the integration of PEG peaks and PCE peaks to deduce the PEG block length (See Table 1).

Synthesis of SPCEs required one last step involving heterogeneous hydrogenation of the alkene moieties, to obtain a structure that closely mimics the PPG block of a poloxamer. Because of the versatility of the unsaturated PCE structure, that can be functionalized/cross‐linked for various applications, we studied the self‐assembly and interfacial properties of both unsaturated PCE‐PEG and saturated SPCE‐PEG. SPCE‐PEG was obtained through the hydrogenation of the respective PCE‐PEG in methanol over palladium on carbon under a hydrogen atmosphere. The block copolymers were purified by dialysis against deionized water. The reaction conversion was monitored by ^1^H NMR, and full conversion was confirmed through the disappearance of the alkene peak. CD confirmed that the PCE retained its helicity after the hydrogenation (See Figure S5, Supporting Information). In total, nine block copolymers were synthesized, with either diblock or triblock architectures, atactic or isotactic tacticity, saturated or unsaturated 6‐membered rings, and varying PEG lengths (100 or 200 repeating units) (See Table 1).

### 2.2 Self‐Assembly Properties of PEG‐Polycycloethers

**Figure 5:**
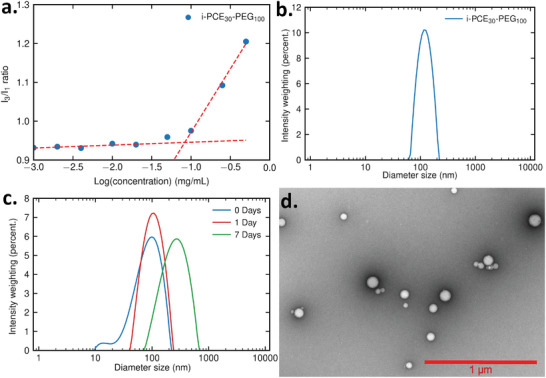
a) CAC plot for isotactic PCE_30_‐*b*‐PEG_100_ using the intensity of the emission spectrum at 373 nm (I_1_) and 384 nm (I_3_). Intensity‐weighted nanoparticle size distributions of b) isotactic PCE_30_‐*b*‐PEG_100_ after a day and c) isotactic SPCE_30_‐*b*‐PCE_30_ over 7 days measured by dynamic light scattering (DLS) in deionized water. d) Transmission electron microscopy (TEM) images of isotactic PCE_30_‐*b*‐PEG_100_ particles formed in deionized water (1 µm scale bar).

**Figure 6:**
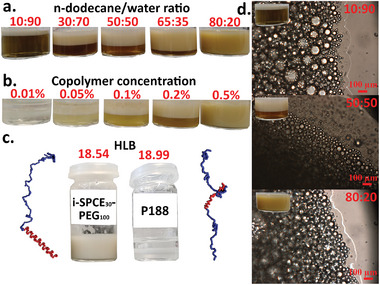
Emulsion pictures of isotactic PCE_30_‐*b*‐PEG_100_ with a) 0.5% block copolymer concentration and a varying oil/water ratio and b) fixed 65:35 oil/water ratio and a varying block copolymer concentration. c) Emulsion pictures and calculated HLB values of isotactic SPCE_30_‐*b*‐PEG_100_ and P188. d) Microscopy images of isotactic PCE_30_‐*b*‐PEG_100_ with a 0.5% block copolymer concentration and a varying oil/water ratio (scale bar 100 µm).

Poloxamers are amphiphilic polymers that can self‐assemble in water. Self‐assembly is driven by the minimization of interactions between the hydrophobic block and water, to reach an equilibrium and reduce the free energy of the system.^[^ ^38^ ^]^ Our PEG‐PCE block copolymers have the same number of carbon and oxygen atoms per block as a poloxamer. The HLB of a poloxamer should be almost identical to its corresponding PEG‐PCE block copolymer when calculated using either Griffin's method or Davies’ method.^[^ ^39^ ^]^ While the main driving force of block copolymer self‐assembly is the relative hydrophobicity of each block, the rigidity of a block can have an influence on the conformations it can take, thus affecting the final morphology of the aggregates.^[^ ^40^, ^41^, ^42^, ^43^, ^44^, ^45^, ^46^ ^]^ In that regard, we decided to study the self‐assembly properties of PEG‐PCE block copolymers in water, to identify any potential divergence from the properties of traditional poloxamers. We first measured the critical aggregation concentration (CAC) of our polymers by fluorescence spectroscopy using pyrene as a fluorescent probe^[^ ^47^ ^]^ (See **Figure** **5**a). For reference, P188 is a PEG_38_‐*b*‐PPG_29_‐*b*‐PEG_38_ block copolymer, it has a similar molecular weight and hydrophilic/hydrophobic ratio as PEG_100_‐*b*‐PCE_30_ (See **Figure** **6**c; Table S3, Supporting Information). The CMC of P188 is published as 4.1 mg mL^−1^ at 37 °C (it increases as the temperature decreases, with no detectable CMC at 20 °C, See Figures S15 and S18, Supporting Information). By comparison, all of our PEG‐PCE block copolymers had a CAC more than ten times lower at 20 °C (see Table 1). Therefore, using a polycyclic structure as a hydrophobic block, one can obtain a block copolymer with a much lower CAC while preserving a short hydrophobic block length. The resulting polymer is then both highly hydrophilic and self‐assembles at low concentrations. A similar result would not be achievable using a poloxamer structure, because the length of the hydrophobic block would have to be increased or the length of the hydrophilic block decreased to obtain a lower CAC (See Figures S15 and S18, Supporting Information).

After exploring the effect of a polycyclic hydrophobic block on the CAC of the block copolymer, we investigated the effect of tacticity. Comparatively, the effect of tacticity (atactic/isotactic), PEG length (100 units or 200 units), architecture (diblock/triblock), and degree of unsaturation had a rather small influence on the value of the block copolymer CAC (See Table 1). Yet, some trends can be noticed: a longer PEG length leads to a higher CAC, triblock copolymers tend to have a lower CAC, isotactic PCEs favor slightly higher CACs, and the presence of unsaturated units did not lead to any noticeable effect on the CAC. The size of the aggregates formed by the self‐assembly of PEG‐PCE block copolymers was measured using dynamic light scattering (DLS) (See Figure 5b). P188 has been reported to form micelles with a 5–10 nm diameter^[^ ^48^ ^]^ and P123 5.0 mg mL^−1^ solution formed self‐assemblies with an average diameter size of 18 nm at 20 °C (See Figure S15, Supporting Information). PEG‐PCE block copolymers formed larger aggregates, with a mean diameter ranging from 64 to 132 nm (See Table S2, Supporting Information). Looking at the intensity distribution of the aggregate diameter size plot, two populations of aggregate can sometimes be observed if the sample is analyzed shortly after preparation, with one major peak ≈100 nm and a smaller peak at 10 nm (See Figure 5c). Considering the small molecular weight of the synthesized block copolymer, this result suggests that the polymer can self‐assemble into 10 nm micelles but will form larger aggregates over time. Again, previously mentioned parameters (tacticity, PEG length, architecture, degree of unsaturation) had a relatively low influence on the size of the aggregates. Samples with lower CAC produced slightly larger aggregates (atactic, shorter PEG, triblock). The main factor leading to larger aggregates is time: The peak at 10 nm was not noticeable by DLS one week after suspending the polymer in water. The mean diameter size also increased over time (See Figure 5c). To confirm the aggregate structure further, the aggregates were imaged using transmission electron microscopy (TEM). The diameter of the aggregates imaged by microscopy correlated with the results from DLS measurements. TEM also indicated the spherical shapes of the aggregates (See Figure 5d). Additional DLS characterization was realized to measure the particle size after 6 months. In intensity weighted distributions a small amount of large particles (500–900 nm) could be observed but the samples consisted mostly of 40–50 nm particles (See Figure S14, Supporting Information), showcasing the stability of the self‐assemblies. In conclusion, tweaking the rigidity of the hydrophobic block of a poloxamer by using a PCE led to a block copolymer with a high hydrophilic/hydrophobic ratio, and a high propensity to form aggregates. This could lead to novel drug delivery systems with high encapsulation efficiencies and high‐loading content.

### 2.3 Emulsification Properties of PEG‐Polycycloethers

Poloxamers are extensively used in the cosmetic industry due to their surfactant properties. For example, poloxamer P184 can be used as an emulsifier for the formulation of various skincare or haircare products like face creams or shampoos. In this regard, the emulsification properties of the synthesized PEG‐PCE block copolymers were studied and compared to traditionally used poloxamers. Despite PEG‐PCE polymers having a similar HLB value as poloxamers, they were expected to have a different behavior as it has been previously reported that the rigidity of a surfactant can influence its properties.^[^ ^49^, ^50^, ^51^ ^]^ To determine what polymer concentration should be used, a batch of emulsions was prepared with an increasing concentration from 0.01 to 0.5 wt.%, and a fixed n‐dodecane/water ratio of 1:1. The polymer used was an isotactic PCE_30_‐*b*‐PEG_100_. A concentration as low as 0.05% was sufficient to obtain an emulsion but it was only at a concentration of 0.5% that a single emulsion phase was obtained (See Figure 6b). Kolliphor P407 technical information recommends a polymer concentration ranging from 1% to 5% to be used as an emulsifier. Thus, a concentration of 0.5% can be considered advantageously low.

For our further studies, all other emulsions were investigated at a fixed polymer concentration of 0.5 wt.%. The second parameter studied was the oil/water ratio with an increasing n‐dodecane content from 10 to 80 vol% (See Figure 6a). All emulsions obtained were oil‐in‐water (O/W) emulsions irrespective of the oil/water ratio. The nature of the emulsion was confirmed by a droplet test, putting a drop of the emulsion in pure n‐dodecane or water to see in which phase it readily dissolves (See Video S1, Supporting Information). The propensity of the block copolymers to form O/W emulsions can be attributed to their high ethylene glycol (EG) content and high HLB value. These O/W emulsions can also be observed using poloxamers with similar molecular weights that have a high EG content like poloxamers P188 or P407. However, using these poloxamers, a high oil content usually leads to an unstable O/W emulsion with phase separation of both the oil and water phases (See Figure 6c). When a PCE and a low oil content were used, a phase separation could be observed over time, with an O/W emulsion phase at the top and a water phase at the bottom. The intensity of this separation decreased with an increasing oil content. The reason seems to be that HIPEs are formed regardless of the oil content used, with excess water forming a separate phase to enable HIPE formation. A single‐phase emulsion, stable for at least 1 week could be obtained using a high oil/water ratio of 80% oil (See Figure 6a). It is worth noting that replacing n‐dodecane with toluene, a low‐viscosity solvent, that is more prone to phase separation, led to the same behavior (See Figure S12, Supporting Information). The HIPE nature of the emulsion was confirmed by the presence of more than 74% oil in the O/W emulsion. Microscopy of the emulsions at different ratios gave similar images of crowded oil droplets, with barely any noticeable continuous phase between them (See Figure 6d; Figures S10 and S11, Supporting Information). The diameter of the droplets was similar throughout the samples, averaging at 61 µm with a standard deviation of 15 µm (See Figure S13, Supporting Information).

Finally, the influence of the block copolymer structure was studied. In particular, the tacticity (atactic/isotactic), the block architecture (diblock/triblock), the PEG length (100 units or 200 units), and the degree of unsaturation in the PCE backbone. A batch of emulsion was prepared with a fixed block copolymer concentration of 0.5% and a fixed oil/water ratio of 65:35, using all of our block copolymers (See Figure S8, Supporting Information). While an oil/water ratio of 80:20 yielded more stable emulsions, we chose an oil/water ratio of 65:35 to check if a phase separation would happen over time for all samples. As mentioned before, the degree of unsaturation on the PCE did not influence the CAC and the self‐assembly of the block copolymer. In a similar fashion, the degree of unsaturation did not seem to have an impact on the emulsification properties of a given PEG‐PCE block copolymer: O/W HIPEs were obtained (See Figures S10 and S11, Supporting Information) and iSPCE_30_‐*b*‐PEG_100_ produced emulsions with the same stability as isotactic PCE_30_‐*b*‐PEG_100_ emulsions. The lack of different behavior of both polymer types could be expected due to the only marginal change in polarity between unsaturated and saturated repeating units. The effective stabilization of emulsions by PCEs with unsaturated units proves itself to be interesting as it might be useful for further design for specific applications, either through functionalization or cross‐linking of the double bond.

Using an isotactic PCE gave a higher CAC, which correlated with less stable emulsions. However, using both an isotactic PCE and a longer PEG chain (200 units) produced stable emulsions. One reason could be that short PEG and isotactic PCE are more prone to self‐aggregation; using a longer PEG would reduce the tendency of the block copolymer to self‐assemble and promote interfacial assemblies. For atactic PCEs, increasing the number of repeating units of the PEG chain from 100 to 200 decreased the CAC of the block copolymer, but it did not drastically change the nature or stability of the emulsions formed. Emulsions formed with the triblock copolymers were less stable, with a phase separation of both water and n‐dodecane after a few days. The effect was more pronounced using an isotactic PCE. Overall, the influence of the isotactic block on the emulsification properties was more pronounced for the triblock copolymer (leading to unstable emulsions unless longer PEG chains were used), while for diblock copolymers the effect of tacticity on emulsion behavior was only marginal. A hypothesis could be that the rigidity of the hydrophobic middle block due to helicity would prevent the bending of the block copolymer into a curved conformation, which is required for efficient interaction of triblock copolymers at oil/water interfaces.

The stability of the emulsions formed was assessed by looking at any phase separation or creaming after 4 months (See Figures S8 and S9, Supporting Information) and inspecting the droplet aspect and size by optical microscopy (See Figure S16 and Table S5, Supporting Information). Atactic PCE_30_‐*b*‐PEG_100_ and atactic SPCE_30_‐*b*‐PEG_100_ showed the best results, with no phase separation and microscopy images still showing HIPEs with a droplet size remaining similar over 6 months.

In order to evaluate our poloxamer mimicking polymer systems with respect to their role models, P188, P407, and P123, various properties (CAC, particle size, surface tension, HIPE formation) were studied for a direct comparison (See Figures S15, S17, and S18 and Table S6, Supporting Information). It was observed that lowering the ethylene oxide content of the block copolymer led to a lower CMC, larger particles, lower surface tensions, and smaller droplet size. It is then interesting to notice that PEG‐PCE behaved more like a poloxamer with a large hydrophobic block than the poloxamer it is structurally mimicking. Even compared to P123, PEG‐PCEs generally showed lower surface tensions, lower CAC, large particle size, and a high propensity to form HIPEs.

Overall, while the HLB of a surfactant is often used to predict the nature of the emulsion formed, the current study shows that the use of a polycyclic hydrophobic block with similar HLB but different rigidity can drastically alter the behavior of a surfactant: lowering its CAC, modifying its self‐assembly morphology and promoting the formation of high‐internal phase emulsions.

## 3 Conclusion

As the demand for high‐performance surfactants increases, the new PEG‐polycycloether block copolymers described here stand as potent alternatives to more traditional poloxamers. They exhibit properties that could not be obtained simply by tuning poloxamer block lengths or end‐groups, for example low CAC with high PEG content, and HIPEs in a broad range of conditions. While additional studies are required to introduce our new polymers into practical applications, these properties could lead to the development of new emulsifiers for use in the food industry, cosmetics, or biomedicine. The impact of a polycyclic middle block highlights the limitations of HLB theory, and further studies are required to understand the explicit relationship between the polycyclic structure and the stereochemistry of the polymers on their conformation in water and at interfaces. The olefin present in the unsaturated polycycloethers allows for functionalization or cross‐linking, paving the way for the preparation of smart surfactants with stimuli‐responsive properties.

## Conflict of Interest

The authors declare no conflict of interest.

## Supporting information

Supporting Information

Supplemental Video 1

## Data Availability

The data that support the findings of this study are available from the corresponding author upon reasonable request.

## References

[1] I. Schmolka (BASF Corp), US3740421A, 1973.

[2] E. Russo , C. Villa , Pharmaceutics 2019, 11, 671. 31835628 10.3390/pharmaceutics11120671 PMC6955690

[3] P. Zarrintaj , J. D. Ramsey , A. Samadi , Z. Atoufi , M. K. Yazdi , M. R. Ganjali , L. M. Amirabad , E. Zangene , M. Farokhi , K. Formela , M. R. Saeb , M. Mozafari , S. Thomas , Acta Biomater. 2020, 110, 37. 32417265 10.1016/j.actbio.2020.04.028

[4] G. Niu , F. Du , L. Song , H. Zhang , J. Yang , H. Cao , Y. Zheng , Z. Yang , G. Wang , H. Yang , S. Zhu , J. Controlled Release 2009, 138, 49. 10.1016/j.jconrel.2009.04.026 19409430

[5] F. Agnely , A. Djedour , A. Bochot , J.‐L. Grossiord , J. Drug Deliv. Technol. 2006, 16, 3.

[6] A. M. Bodratti , P. Alexandridis , J. Funct. Biomater. 2018, 9, 11. 29346330 10.3390/jfb9010011 PMC5872097

[7] https://www.precisionreports.co/global‐poloxamer‐market‐19882483 (accessed: October 2023).

[8] S. D. Singh‐Joy , V. C. McLain , Int. J. Toxicol. 2008, 27, 93. 18830866 10.1080/10915810802244595

[9] E. H. Gökçe , E. A. Yapar , S. T. Tanrıverdi , Ö. Özer , in Nanobiomaterials in Galenic Formulations and Cosmetics (Ed.: A.M. Grumezescu ), William Andrew Publishing, Norwich, NY, 2016, p. 363.

[10] B. Mazumder , S. Ray , P. Pal , Y. Pathak , in Nanotechnology: Therapeutic, Nutraceutical, and Cosmetic Advances, CRC Press, Boca Raton, FL, 2019.

[11] E. Esposito , M. Drechsler , P. Mariani , E. Sivieri , R. Bozzini , L. Montesi , E. Menegatti , R. Cortesi , Int. J. Cosmet. Sci. 2007, 29, 39. 18489310 10.1111/j.1467-2494.2007.00362.x

[12] O. Šrom , V. Trávníková , L. Bláha , M. Ciofalo , M. Šoóš , Biochem. Eng. J. 2022, 186, 108549.

[13] P. Lemieux , N. Guérin , G. Paradis , R. Proulx , L. Chistyakova , A. Kabanov , V. Alakhov , Gene Ther. 2000, 7, 986. 10849559 10.1038/sj.gt.3301189

[14] H. Madry , L. Gao , A. Rey‐Rico , J. K. Venkatesan , K. Müller‐Brandt , X. Cai , L. Goebel , G. Schmitt , S. Speicher‐Mentges , D. Zurakowski , M. D. Menger , M. W. Laschke , M. Cucchiarini , J. Adv. Mater. 2020, 32, 1906508. 10.1002/adma.201906508 31763733

[15] L. J. Feldman , C. J. Pastore , N. Aubailly , M. Kearney , D. Chen , M. Perricaudet , P. G. Steg , J. M. Isner , Gene Ther. 1997, 4, 189. 9135732 10.1038/sj.gt.3300382

[16] H. Peng , K. M. Hall , B. Clayton , K. Wiltberger , W. Hu , E. Hughes , J. Kane , R. Ney , T. Ryll , Biotechnol. Prog. 2014, 30, 1411. 25098761 10.1002/btpr.1967

[17] T. K. Law , T. L. Whateley , A. T. Florence , Int. J. Pharm. 1984, 21, 277.

[18] M. A. Abou‐Shamat , J. Calvo‐Castro , J. L. Stair , M. T. Cook , Macromol. Chem. Phys. 2019, 220, 1900173.

[19] L. Ci , Z. Huang , Y. Liu , Z. Liu , G. Wei , W. Lu , Acta Pharm. Sin. B. 2017, 7, 593. 28924553 10.1016/j.apsb.2017.03.002 PMC5595263

[20] A. M. Butt , M. C. I. Mohd Amin , H. Katas , Int. J. Nanomed. 2015, 10, 1321. 10.2147/IJN.S78438 PMC4335624 25709451

[21] M. Worm , B. Kang , C. Dingels , F. R. Wurm , H. Frey , Macromol. Rapid Commun. 2016, 37, 775. 27000789 10.1002/marc.201600080

[22] L. Wang , J. Yao , X. Zhang , Y. Zhang , C. Xu , R. J. Lee , G. Yu , B. Yu , L. Teng , Colloids Surf., B 2018, 161, 464. 10.1016/j.colsurfb.2017.11.013 29128832

[23] C. Yu , Y. Yu , D. Zhao , Chem. Commun. 2000, 36, 575.

[24] S. W. Mork , G. D. Rose , D. P. Green , J. Surfactants Deterg. 2001, 4, 127.

[25] O. Marin , M. Alesker , S. Guttman , G. Gershinsky , E. Edri , H. Shpaisman , R. E. Guerra , D. Zitoun , M. Deutsch , E. Sloutskin , J. Colloid Interface Sci. 2019, 538, 541. 30551067 10.1016/j.jcis.2018.11.111

[26] C. F. Welch , G. D. Rose , D. Malotky , S. T. Eckersley , Langmuir 2006, 22, 1544. 16460072 10.1021/la052207h

[27] H. Gao , L. Ma , C. Cheng , J. Liu , R. Liang , L. Zou , W. Liu , D. J. McClements , Trends Food Sci. Technol. 2021, 112, 36.

[28] N. R. Cameron , Polymer 2005, 46, 1439.

[29] I. Pulko , P. Krajnc , Macromol. Rapid Commun. 2012, 33, 1731. 22907672 10.1002/marc.201200393

[30] L. L. C. Wong , P. M. Baiz Villafranca , A. Menner , A. Bismarck , Langmuir 2013, 29, 5952. 23617331 10.1021/la3047643

[31] J. Luo , Z. Huang , L. Liu , H. Wang , G. Ruan , C. Zhao , F. Du , J. Sep. Sci. 2021, 44, 169. 32845083 10.1002/jssc.202000612

[32] M. Alkattan , J. Prunet , M. P. Shaver , Angew. Chem., Int. Ed. 2018, 57, 12835. 10.1002/anie.201805113 PMC6175094 29873428

[33] G. Settanni , J. Zhou , F. Schmid , J. Phys.: Conf. Ser. 2017, 921, 012002.

[34] X. Liu , G. Sala , E. Scholten , Curr. Res. Food. Sci. 2023, 7, 100531. 37441167 10.1016/j.crfs.2023.100531 PMC10333429

[35] D. E. White , P. M. Tadross , Z. Lu , E. N. Jacobsen , Tetrahedron 2014, 70, 4165. 25045188 10.1016/j.tet.2014.03.043 PMC4096935

[36] A. D. Adler , F. R. Longo , J. D. Finarelli , J. Goldmacher , J. Assour , L. Korsakoff , J. Org. Chem. 1967, 32, 476.

[37] K. Philipps , T. Junkers , J. Michels , Polym. Chem. 2021, 12, 2522.

[38] J. Szafraniec , A. Antosik , J. Knapik‐Kowalczuk , K. Chmiel , M. Kurek , K. Gawlak , J. Odrobińska , M. Paluch , R. Jachowicz , Pharmaceutics 2019, 11, 130. 30893859 10.3390/pharmaceutics11030130 PMC6470807

[39] R. Pasquali , N. Sacco , C. Bregni , Lat. Am. J. Pharm. 2009, 28, 313.

[40] E. C. Davidson , A. M. Rosales , A. L. Patterson , B. Russ , B. Yu , R. N. Zuckermann , R. A. Segalman , Macromolecules 2018, 51, 2089.

[41] B. D. Olsen , R. A. Segalman , Mater. Sci. Eng. R Rep. 2008, 62, 37.

[42] B. Yu , S. P. O. Danielsen , A. L. Patterson , E. C. Davidson , R. A. Segalman , Macromolecules 2019, 52, 2560.

[43] R.‐M. Ho , C.‐K. Chen , Y.‐W. Chiang , Macromol. Rapid Commun. 2009, 30, 1439. 21638404 10.1002/marc.200900181

[44] Y.‐W. Chiang , R.‐M. Ho , C. Burger , H. Hasegawa , Soft Matter 2011, 7, 9797.

[45] R.‐M. Ho , Y.‐W. Chiang , S.‐C. Lin , C.‐K. Chen , Prog. Polym. Sci. 2011, 36, 376.

[46] A. Halperin , Macromolecules 1990, 23, 2724.

[47] H. Li , D. Hu , F. Liang , X. Huang , Q. Zhu , R. Soc. Open Sci. 2020, 7, 192092. 32269815 10.1098/rsos.192092 PMC7137975

[48] https://pharma.basf.com/technicalinformation/30631536/kolliphor‐p‐188‐geismar (accessed: October 2023).

[49] M. A. Schafheutle , H. Finkelmann , Liq. Cryst. 1988, 3, 1369.

[50] B. D. Marshall , K. R. Cox , W. G. Chapman , Soft Matter 2012, 8, 7415.

[51] J. A. Hanson , C. B. Chang , S. M. Graves , Z. Li , T. G. Mason , T. J. Deming , Nature 2008, 455, 85. 18769436 10.1038/nature07197

