# Supplementary material for: Synthesis of PEG‐Polycycloether Block Copolymers: Poloxamer Mimics Containing a Rigid Helical Block
Source: Adv Sci (Weinh). 2024 Mar 23;11(22):2310277. doi: 10.1002/advs.202310277 (PMC11165552; doi:10.1002/advs.202310277)
Supplement: Supplementary file 1 — Supporting Information [file ADVS-11-2310277-s002.pdf]

ADVANCED SCIENCE
Open Access

Supporting Information

for *Adv. Sci.*, DOI 10.1002/advs.202310277

Synthesis of PEG-Polycycloether Block Copolymers: Poloxamer Mimics Containing a Rigid Helical Block

Jean-Baptiste Masclef, Joëlle Prunet and Bernhard V. K. J. Schmidt**

Supporting Information

Synthesis of PEG-Polycycloether Block Copolymers: Poloxamer Mimics Containing a Rigid Helical Block

Jean-Baptiste Masclef, Joëlle Prunet, Bernhard V. K. J. Schmidt

School of Chemistry, University of Glasgow, Joseph Black Building, G12 8QQ, Glasgow, UK

Email: joelle.prunet@glasgow.ac.uk

Email: bernhard.schmidt@glasgow.ac.uk

Experimental part

Materials

Benzaldehyde (≥99%), calcium hydride, propionic acid, n-dodecane and pyrrole (98%) were obtained from Alfa Aesar. Diethylaluminum chloride solution in hexane (1 M), ethylene oxide solution in THF (2.5 M), lithium aluminum hydride solution in THF (1 M), palladium on activated carbon (10% wt), silver nitrate (≥99%) and sodium hydride were obtained from Sigma-Aldrich. Grubbs II catalyst (≥97%) was purchased from Carbosynth. (−)-(*R,R*)-*N,N'*-*bis*(3,5-di-*tert*-Butylsalicylidene)-1,2-cyclohexanediaminocobalt(II) (98%), acetic acid (≥99%) and 1,2-dichloroethane (99.8%, extra dry) were obtained from Fisher Scientific. Tetrahydrofuran (THF), dichloromethane (DCM), and toluene were obtained using an in-house solvent purification system (Pure-SolvTM 500 Solvent Purification System). Other solvents were purchased from Fisher chemicals and were HPLC-grade.

Butadiene monoxide (Alfa Aesar, 98%) was dried over calcium hydride for 24 h under reflux and distilled under an atmosphere of argon. Reactions involving air-sensitive agents and dry solvents were performed in glassware that had been oven-dried (150 °C) or flame-dried prior to use. These reactions were carried out with the exclusion of air using an argon atmosphere. Preparative size-exclusion chromatography (SEC) was performed under forced flow conditions using HPLC graded solvents and Sephadex LH-20 (Alfa Aesar) as solid support. Tetraphenylporphyrin ($TPPH_2$) was synthesized according to literature procedures.[48] Rochelle salt solution (10%) was prepared by dissolving potassium sodium tartrate tetrahydrate (Sigma-Aldrich) in deionized water.

Methods

Block copolymer synthesis

Synthesis of enantiopure butadiene monoxide

According to the literature,[33] enantioenriched butadiene monoxide was obtained by hydrolytic kinetic resolution using a chiral (salen)Co^{III} complex. The commercially available pro-ligand (*R*,*R*)-*N,N'-bis*(3,5-di-*tert*-butylsalicylidene)-1,2-cyclohexanediaminocobalt(II) (1.3 g, 2.1 mmol, 0.015 equiv) in toluene (10 mL) was treated with acetic acid (1.5 mL) and stirred under air for 3 h. The crude mixture was left under vacuum overnight. The complex residue obtained was dissolved in racemic 3,4-epoxy-1-butene (10.0 g, 140 mmol). The solution was cooled to 0 °C and deionized water (1.80 mL, 100 mmol, 0.71 equiv) was added dropwise. After 96 h at ambient temperature, (*R*)-3,4-epoxy-1-butene (2.50 g, 35.7 mmol, 32%) was isolated by vacuum transfer into a catch flask cooled with liquid N_2.

Typical procedure for the synthesis of chloride end-functionalized poly(epoxybutene) – Cl-PEB$_{30}$

PEB was synthesized according to a modified literature procedure.[32] TPPAlCl was prepared in a flame dried Schlenk tube by dissolving $TPPH_2$ (1.35 g, 2.19 mmol, 1 equiv) in DCM (40 mL) and adding a 1 M diethylaluminum chloride solution in hexane (1 equiv) slowly. After 3 h the volatiles were evaporated using vacuum and a nitrogen cooled trap. TPPAlCl was then dried overnight. Racemic or isotactic 3,4-epoxy-1-butene (4.20 g, 4.83 mL, 60.0 mmol, 30 equiv) was added the next day to the dried (TPP)AlCl and the resulting mixture was subsequently stirred at ambient temperature for 2 days. The mixture was quenched by adding 1 M aqueous HCl (2 mL) under stirring. The volatiles were removed, and the resulting polymer was dissolved in a 1:1 methanol/DCM mixture for purification by preparative SEC (Sephadex LH-20, methanol/DCM

1:1). The fractions were concentrated, and the polymer was dried under vacuum overnight to give a brown oil (atactic Cl-PEB_{30}: 3.44 g, 82%).

Typical procedure for the reduction of poly(epoxybutene) chloride end-group leading to H-PEB_{30}

Atactic or isotactic H-PEB_{30} was synthesized by dissolving the corresponding Cl-PEB_{30} (400 mg, 190 µmol, 1 equiv) in THF (4 mL), then slowly adding a $LiAlH_4$ solution in THF (1 M, 2.0 mL, 2.0 mmol, 10 equiv). The mixture was stirred at ambient temperature for 48 h, quenched with methanol (1 mL) then concentrated under vacuum. DCM (10 mL) and a 10% aqueous Rochelle salt solution (10 mL) were added and the mixture was stirred for 4 h. The organic phase was then separated, washed three times with deionized water, dried over magnesium sulfate, and filtered. The volatiles were removed under vacuum, and the resulting polymer was dissolved in a 1:1 methanol/DCM mixture for purification by preparative SEC (Sephadex LH-20, methanol/DCM 1:1). The fractions were concentrated, and the polymer was dried under vacuum overnight to give a brown oil (atactic H-PEB_{30}: 324 mg, 81%).

Typical procedure for the hydroxy substitution of poly(epoxybutene) chloride end-group leading to HO-PEB_{30}

Atactic or isotactic HO-PEB_{30} was synthesized by dissolving the corresponding Cl-PEB_{30} (500 mg, 240 µmol, 1 equiv) and silver nitrate (210 mg, 2.50 mmol, 10 equiv) in a mixture of ethanol (10 mL) and deionized water (2 mL). The reaction mixture was then refluxed for 48 h and subsequently concentrated under vacuum. The resulting polymer was dissolved in DCM (10 mL), and the resulting solution washed three times with water, dried over magnesium sulfate, and

filtered. The volatiles were removed under vacuum, and the resulting polymer was dissolved in a 1:1 methanol/DCM mixture for purification by preparative SEC (Sephadex LH-20, methanol/DCM 1:1). The fractions were concentrated, and the polymer was dried under vacuum overnight to give a brown oil (atactic HO-PEB_{30}: 430 mg, 86%).

Typical procedure for the synthesis of polycycloether – PCE_{30}

Isotactic or atactic PCE_{30} was synthesized according to a modified literature procedure.[32] In a large round-bottomed flask, atactic or isotactic H-PEB_{30} or HO-PEB_{30} (600 mg, 8.57 mmol) was stirred for 15 min at 84 °C in DCE (38 mL). Then, second-generation Grubbs catalyst (178 mg, 210 µmol, 2.5 mol%) in DCE was added slowly under argon. After 5 days, the reaction mixture was cooled to ambient temperature, treated with 100 equiv of DMSO and stirred for 1 h. The volatiles were removed under vacuum, and the residue was purified using preparative SEC (Sephadex LH-20, 3:1 methanol/DCM). The volatiles were removed under vacuum, and the resulting PCE was dried overnight (atactic H-PCE_{30}: 398 mg, 83%).

Typical procedure for the synthesis of polycycloether-block-poly(ethylene glycol) – PCE_{30}-b-PEG_{100}

In a flame-dried Schlenk tube, atactic or isotactic H-PCE_{30} (300 mg, 0.14 mmol, 1 equiv) was added and dried overnight under vacuum. Sodium hydride was added (36 mg, 1.5 mmol, 10 equiv), dry THF was introduced (3 mL), then the mixture was stirred at 50 °C for 3 h. The mixture was cooled down to ambient temperature then ethylene oxide in THF (2.5 M, 6.0 mL, 15 mmol, 100 equiv) was added and the mixture was stirred at 50 °C for 2 days. An aqueous sodium hydroxide solution was then added (1 M, 1.0 mL) and the mixture was stirred overnight at ambient temperature. The mixture was finally quenched with an aqueous HCl solution (1 M, 1.0 mL) then

the volatiles were removed under vacuum. The resulting polymer was dissolved in deionized water and dialyzed (1.0 kDa MWCO) against deionized water over two days. The resulting solution was freeze-dried and PCE_{30}-*b*-PEG_{100} was obtained as a viscous brown oil (atactic PCE_{30}-*b*-PEG_{100}: 522 mg, 57% yield).

Typical procedure for the synthesis of poly(ethylene glycol)-block-polycycloether-block-poly(ethylene glycol) – PEG_{50}-PCE_{30}-b-PEG_{50}

PEG_{50}-*b*-PCE_{30}-*b*-PEG_{50} was synthesized using the previous procedure, using atactic or isotactic HO-PCE_{30} as substrate.

Typical procedure for the synthesis of saturated polycycloether-block-poly(ethylene glycol) – $SPCE_{30}$-b-PEG_{100}

Atactic or isotactic $SPCE_{30}$-*b*-PEG_{100} was synthesized by adding the corresponding PCE_{30}-*b*-PEG_{100} (58 mg) to a 25 mL round-bottom flask. Under an argon atmosphere, methanol was added (2 mL) followed by Pd/C (10% wt, 12 mg). Hydrogen was then introduced to the flask. The mixture was stirred at ambient temperature for 24 h. The reaction mixture was then filtered, concentrated under vacuum, and the resulting oil solubilized in water, followed by dialysis (1.5 kDa MWCO) against water for 24 h. The aqueous solution was freeze-dried and $SPCE_{30}$-*b*-PEG_{100} was obtained as a viscous yellow oil (atactic $SPCE_{30}$-*b*-PEG_{100}: 42 mg, 76%).

Typical procedure for the synthesis of poly(ethylene glycol)-block-saturated poly(cycloether)-block-poly(ethylene glycol) – PEG_{50}-b-$SPCE_{30}$-b-PEG_{50}

PEG_{50}-*b*-$SPCE_{30}$-*b*-PEG_{50} was synthesized using the previous procedure, using atactic or isotactic PEG_{50}-*b*-PCE_{30}-*b*-PEG_{50} as substrate.

Emulsion preparation

To prepare an emulsion, the typical procedure consisted of dissolving the PEG-polycycloether block copolymer in water, adding the oil, keeping a total volume of 4 mL, then using IKA Ultra-Turrax T18 homogenizer for 4 minutes at 13,500 rpm. Toluene, n-dodecane and rapeseed oil were investigated as oil types. Blo9ck copolymer concentration ranged from 0.01% to 0.5%. Oil/water ratios ranged from 5% to 90%. The nature of the emulsion (O/W versus W/O) was determined by measuring the conductivity of the emulsion as well as doing a drop test; that is, putting a drop of the emulsion either in pure oil or pure water (See Supp. Info. Video S1).

Characterization

^{1}H NMR and DOSY spectra were recorded on either a Bruker AVI DPX-400 or a Bruker DPX-400 (400 MHz) instrument. The chemical shifts are expressed in parts per million (ppm) referenced to TMS. D_2O or $CDCl_3$ was used as solvent. **Size-exclusion chromatography (SEC)** was conducted in THF at 35 °C using a column system with an Agilent PL Gel Guard Column (5 µm) and an Agilent PL Gel Mixed-D Column (5 µm) as well as an Agilent Infinity1260 II RID and calibration with poly(styrene) standards. **Transmission electron microscopy (TEM)** experiments were performed on a JEOL 1200 EX TEM running at 80 kV, images were captured using a Cantega 2K X 2K camera and Olympus ITEM Software. To prepare negative stained samples: suspension droplets (5 µL) were placed on top of the surface of carbon coated 400 mesh copper grids that were previously glow discharged using a Quorum Q150T ES High vacuum system. Samples were left for 5 min to allow attachment, then grids were floated sample side down three times for 30 s each onto distilled water droplets before negative staining with 2% aqueous uranyl acetate for 5 min then allowed to air dry before imaging. **High-resolution mass spectrometry (HRMS)** was

performed on a Bruker microTOFq High Resolution Mass Spectrometer using an Electrospray (ESI) ion source coupled to a time-of-flight (ToF) analyzer. **Multi-angle dynamic light scattering (MADLS)** measurements were performed using an Anton Paar Litesizer 500 using forward scattering (15°), side scattering (90°) and back scattering (175°). The light source was a semiconductor laser diode at 40 mW, 658 nm. All experiments were performed three times and the average size distribution was calculated. Emulsion droplets were analyzed using a Leica DM750 binocular **microscope** without any dilution. Size distribution was collected using ImageJ software. Enantiopure butadiene monoxide optical rotation was measured using an Autopol III **Polarimeter**, with a mean value obtained through 5 measurements. **Surface tension** values were measured by using an Ossila contact angle goniometer. Block copolymer solutions in water were prepared with a fixed concentration of 0.5 wt% and analyzed at ambient temperature. For each solution, shape analysis was performed on three drops, using 5 different frames for each drop, the surface tension value was then averaged (See Figure S6 and Table S4). **Hydrophilic-Lipophilic Balance (HLB) values** were calculated using Chemaxon HLB predictor through the MarvinSketch software, using Griffin's method. **Circular dichroism** studies were performed on a Chirascan circular dichroism spectrometer. Temperature was kept at 20 °C, spectra were recorded in triplicates from 180 to 260 nm, with an acquisition time of 1.0 s and a wavelength step increase of 1 nm. The sample concentration was kept at 0.1 mg/mL. The critical aggregation concentration (CAC) of each block copolymer was measured by **fluorescence spectroscopy** using pyrene as a fluorescence probe as described in the literature.[47] Measurements were made on a Horiba Duetta Bio fluorescence and absorbance spectrometer. A blank consisting of deionized water was used before each experiment. Block copolymer solutions of different concentrations were prepared in water, before adding 12 μL of a 0.5 M pyrene solution in ethanol. Both blank and sample absorption spectra were recorded before each experiment to account for the Inner Filter Effect. Excitation wavelength was fixed at

334 nm and emission wavelength was recorded from 360 to 450 nm. Integration time was 0.1 s, detector binning was 0.5 nm (1 pixel). Excitation and emission bandpass were 5 nm. Peak intensity was measured at 372 nm (I_1) and 384 nm (I_3). CAC was determined by plotting the I_3/I_1 ratio against the block copolymer concentration and reading the intersection of the regression trendlines at low and high concentrations.

Statistical Analysis

When **dynamic light scattering** was used, the standard deviation of the intensity-weighted diameter size was calculated using Anton Paar Kalliope particle sizing software.

The root-mean-square (RMS) error associated with **surface tension** values were calculated using Ossila contact angle goniometer software. An RMS error greater than 1 between the detected edge data and the polynomial fit is considered poor.

The standard deviation associated with the **size distribution of emulsion droplets** was calculated using ImageJ software, with a sample size of 100 droplets.

Synthesis of the polymers

Synthesis of tetraphenylporphyrin ((TPP)H_2)

(TPP)H_2 was synthesized from freshly distilled pyrrole (4.6 g, 68 mmol) and benzaldehyde (7.2 g, 68 mmol) in propionic acid (300 mL) under reflux for 4 h. The crude product was precipitated upon standing overnight at room temperature, filtered, and then washed with water and methanol (20 mL). The obtained crystals were recrystallized from $CHCl_3/CH_3OH$ (1:2 v/v) and dried overnight under vacuum to give $TPPH_2$ (2.8 g, 27%) as purple crystals.[36]

The following polymers were synthesized according to the methods described above:

Synthesis of atactic poly(epoxybutene) aCl-PEB$_{30}$

Yield: 3.44 g, 82%

Synthesis of isotactic poly(epoxybutene) iCl-PEB$_{30}$

Yield: 1.21 g, 78%

Reduction of atactic poly(epoxybutene) chloride end-group – aH-PEB$_{30}$

Yield: 324 mg, 81%

Reduction of poly(epoxybutene) chloride end-group – iH-PEB$_{30}$

Yield: 300 mg, 75%

Hydroxy substitution of atactic poly(epoxybutene) chloride end-group – aHO-PEB$_{30}$

Yield: 430 mg, 86%

Hydroxy substitution of isotactic poly(epoxybutene) chloride end-group – iHO-PEB$_{30}$

Yield: 203 mg, 81%

Synthesis of atactic polycycloether aH-PCE$_{30}$

Yield: 398 mg, 83%

Synthesis of atactic polycycloether aHO-PCE$_{30}$

Yield: 166 mg, 89%

Synthesis of isotactic polycycloether iH-PCE$_{30}$

Yield: 155 mg, 82%

Synthesis of isotactic polycycloether iHO-PCE$_{30}$

Yield: 143 mg, 84%

Synthesis of atactic polycycloether-block-poly(ethylene glycol) aPCE$_{30}$-PEG$_{100}$

Yield: 522 mg, 57%

Synthesis of atactic polycycloether-block-polyethylene glycol) aPCE$_{30}$-PEG$_{200}$

Yield: 412 mg, 65%

Synthesis of isotactic polycycloether-block-poly(ethylene glycol) iPCE$_{30}$-PEG$_{100}$

Yield: 315 mg, 62%

Synthesis of isotactic polycycloether-block-poly(ethylene glycol) iPCE$_{30}$-PEG$_{200}$

Yield: 219 mg, 52%

Synthesis of atactic saturated polycycloether-block-poly(ethylene glycol) aSPCE$_{30}$-PEG$_{100}$

Yield: 42 mg, 76%

Synthesis of isotactic saturated polycycloether-block-poly(ethylene glycol) iSPCE$_{30}$-PEG$_{100}$

Yield: 59 mg, 81%

Synthesis of atactic poly(ethylene glycol)-block-polycycloether-block-poly(ethylene glycol) PEG_{50}-aPCE_{30}-PEG_{50}

Yield: 414 mg, 58%

Synthesis of atactic poly(ethylene glycol)-block-saturated polycycloether-block-poly(ethylene glycol) PEG_{50}-a$SPCE_{30}$-PEG_{50}

Yield: 81 mg, 85%

Synthesis of isotactic poly(ethylene glycol)-block-polycycloether-block-poly(ethylene glycol) PEG_{50}-iPCE_{30}-PEG_{50}

Yield: 95 mg, 78%

Mass spectrometry

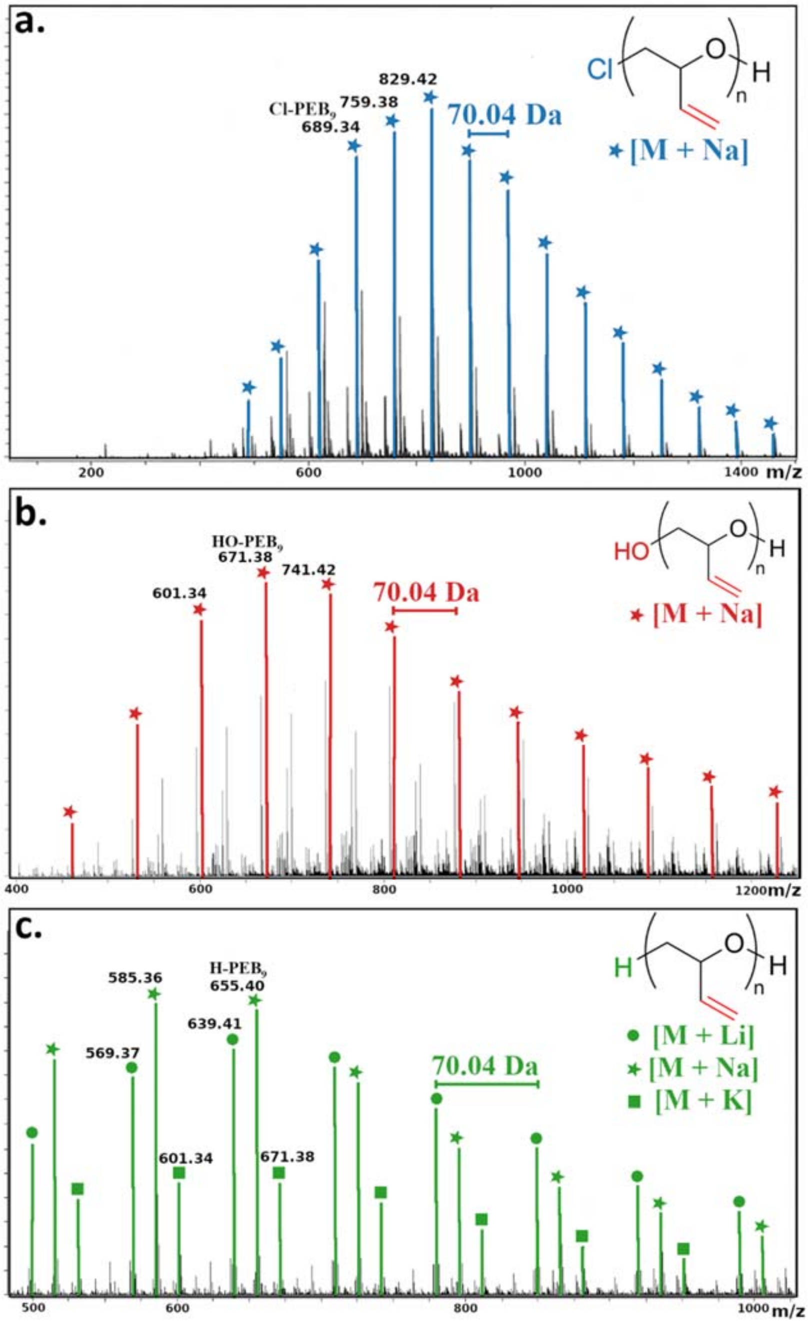

Figure S1. Mass spectra of atactic Cl-PEB15, atactic HO-PEB15, atactic H-PEB15 and corresponding adducts. Adducts peaks are separated by 70.04 Da. The polymers with 9 repeating units have been pinpointed in each spectrum.

Table S1. m/z values of Cl-PEB_{15}, HO-PEB_{15} and H-PEB_{15} adducts. The adducts in bold are shown in Figure S1.

Cl-PEB Adducts	m/z
**[Na + Cl-PEB_8] ***	619.2969
**[Na + Cl-PEB_9] ***	689.3376
**[Na + Cl-PEB_{10}] ***	759.3787
[Na + Cl-PEB_{11}]	829.4189
[Na + Cl-PEB_{12}]	899.4596
[Na + Cl-PEB_{13}]	969.4997
[Na + Cl-PEB_{14}]	1039.5397
[Na + Cl-PEB_{15}]	1109.5798
[Na + Cl-PEB_{16}]	1179.6174
[Na + Cl-PEB_{17}]	1249.6595
[Na + Cl-PEB_{18}]	1319.6997
[Na + Cl-PEB_{19}]	1389.7387
[Na + Cl-PEB_{20}]	1459.7722

HO-PEB Adducts	m/z
**[Na + HO-PEB_8] ***	531.2948
**[Na + HO-PEB_9] ***	601.3386
**[Na + HO-PEB_{10}] ***	671.3822
[Na + HO-PEB_{11}]	741.4258
[Na + HO-PEB_{12}]	811.4692
[Na + HO-PEB_{13}]	881.5127
[Na + HO-PEB_{14}]	951.5563
[Na + HO-PEB_{15}]	1021.5998

**H-PEB Adducts**	**m/z**
[Li + H-PEB$_7$]	499.3253
[Na + H-PEB$_7$]	515.3201
[K + H-PEB$_7$]	531.2937
**[Li + H-PEB$_8$] ***	569.3673
**[Na + H-PEB$_8$] ***	585.3620
**[K + H-PEB$_8$] ***	601.3357
**[Li + H-PEB$_9$] ***	639.4093
**[Na + H-PEB$_9$] ***	655.1037
**[K + H-PEB$_9$] ***	671.3776
[Li + H-PEB$_{10}$]	709.4512
[Na + H-PEB$_{10}$]	725.4452
[K + H-PEB$_{10}$]	741.4195
[Li + H-PEB$_{11}$]	779.4931
[Na + H-PEB$_{11}$]	795.4866
[K + H-PEB$_{11}$]	811.4613
[Li + H-PEB$_{12}$]	849.5348
[Na + H-PEB$_{12}$]	865.5273
[K + H-PEB$_{12}$]	881.5031
[Li + H-PEB$_{13}$]	919.5765
[Na + H-PEB$_{13}$]	935.5677
[K + H-PEB$_{13}$]	951.5451

Size-exclusion chromatography

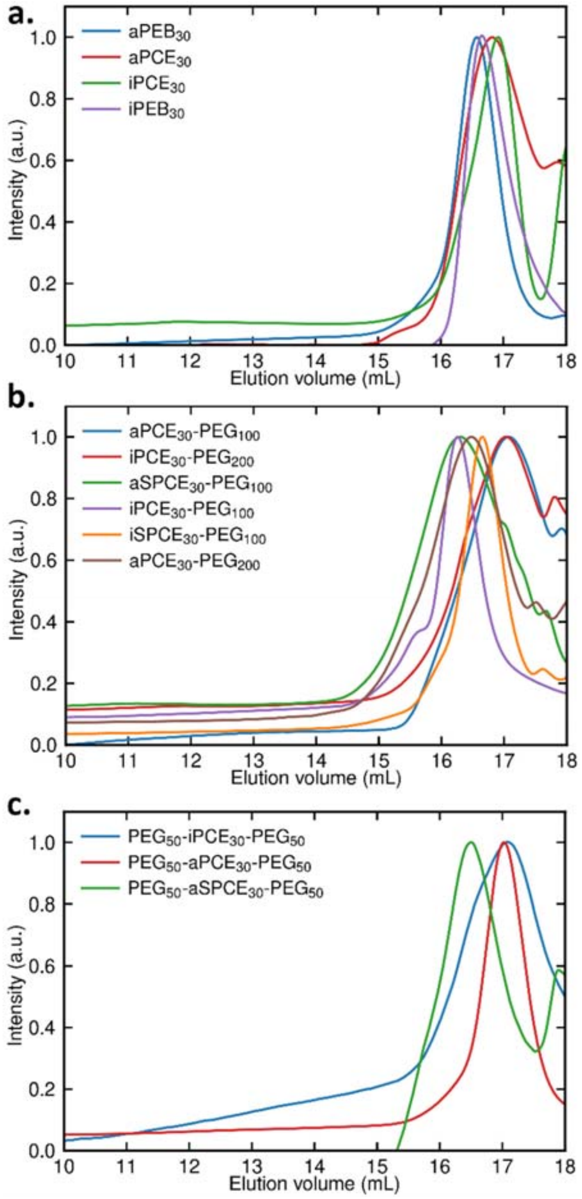

Figure S2. Size-exclusion chromatography traces of **a)** the homopolymers, **b)** diblock and **c)** triblock copolymers measured in THF.

^{1}H NMR

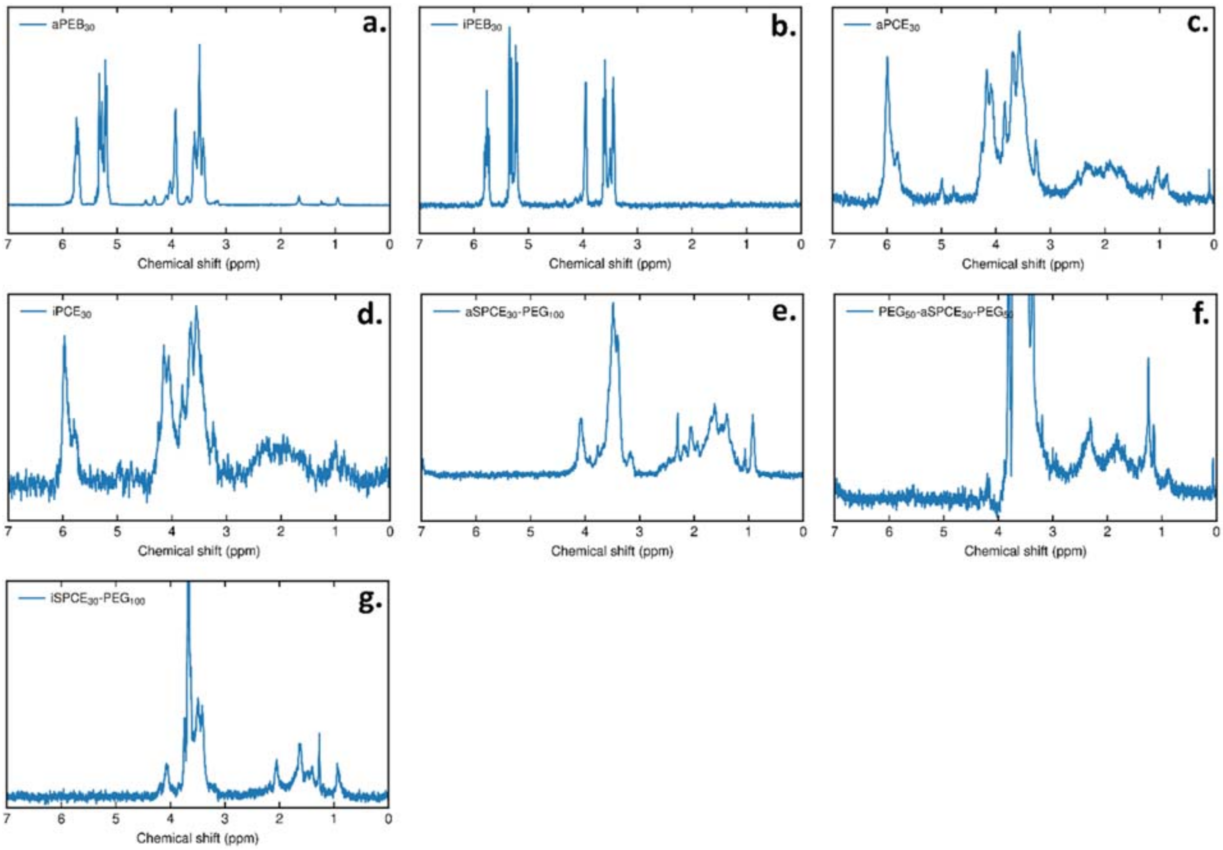

Figure S3. ^{1}H NMR of the synthesized polymers in $CDCl_3$: **a)** aPEB$_{30}$, **b)** iPEB$_{30}$, **c)** aPCE$_{30}$, **d)** iPCE$_{30}$, **e)** aSPCE$_{30}$-PEG$_{100}$, **f)** PEG$_{50}$-aSPCE$_{30}$-PEG$_{50}$ and **g)** iSPCE$_{30}$-PEG$_{100}$.

DOSY

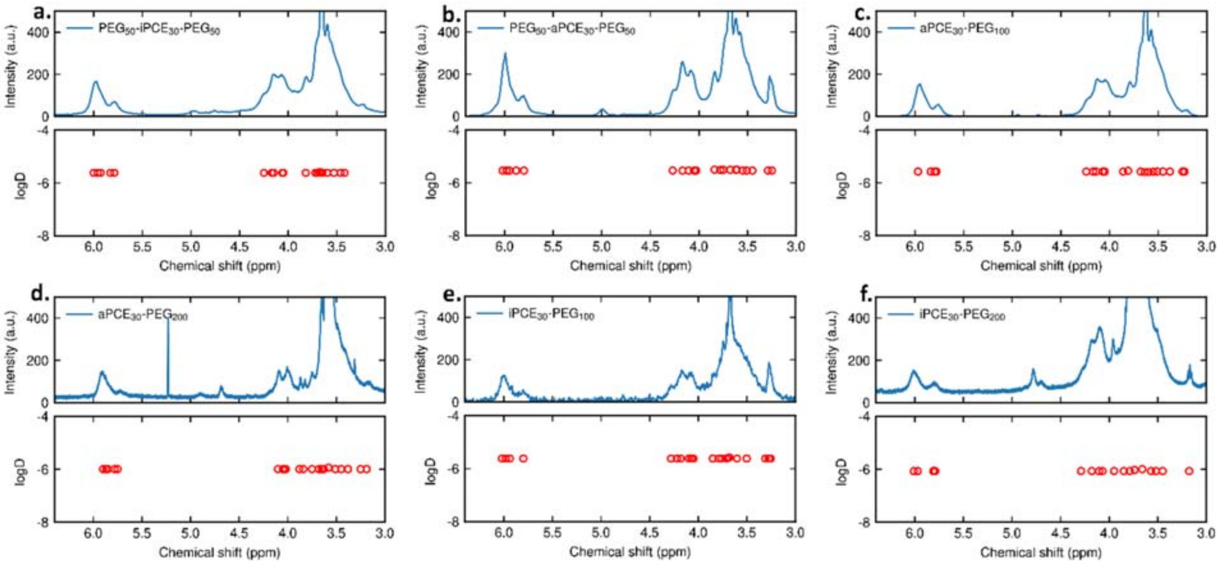

Figure S4. Diffusion-Ordered Spectroscopy (DOSY) ^{1}H NMR in $CDCl_3$: **a)** PEG_{50}-$iPCE_{30}$-PEG_{50}, **b)** PEG_{50}-$aPCE_{30}$-PEG_{50}, **c)** $aPCE_{30}$-PEG_{100}, **d)** $aPCE_{30}$-PEG_{200}, **e)** $iPCE_{30}$-PEG_{100} and **f)** $iPCE_{30}$-PEG_{200}.

Circular dichroism

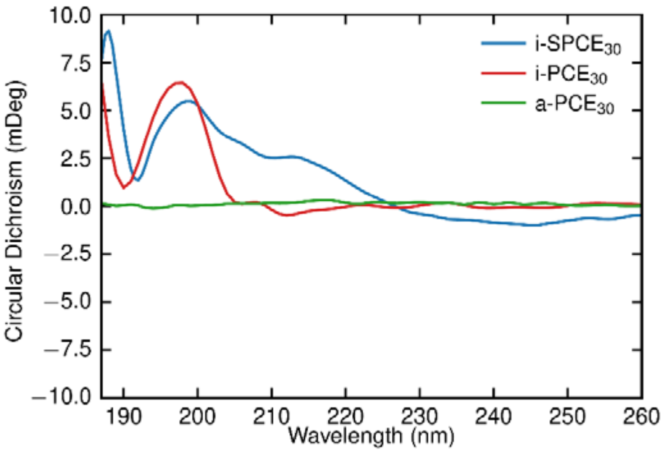

Figure S5. Circular dichroism plot of a-PCE_{30}, i-PCE_{30} and i-$SPCE_{30}$ as a 0.1 mg/mL solution in methanol.

Dynamic light scattering

Table S2. Nanoparticle diameter (intensity weighting) of the synthesized PEG-PCE block copolymers in deionized water (1 mg/mL), measured via dynamic light scattering with a 175° scattering angle.

Block copolymer	Nanoparticle diameter (nm)
$aPCE_{30}$-PEG_{100}	109
$aPCE_{30}$-PEG_{200}	93
$iPCE_{30}$-PEG_{100}	101
$iPCE_{30}$-PEG_{200}	64
$aSPCE_{30}$-PEG_{100}	92
$iSPCE_{30}$-PEG_{100}	85
PEG_{50}-$aPCE_{30}$-PEG_{50}	132
PEG_{50}-$aSPCE_{30}$-PEG_{50}	121
PEG_{50}-$iPCE_{30}$-PEG_{50}	89

HLB values

Table S3. Hydrophilic-Lipophilic Balance (HLB) of the polymer and block copolymer synthesized, calculated through the Griffin method with the ChemSketch software.

Block copolymer	HLB
PEB_{30}	12.08
PCE_{30}	15.20
$aPCE_{30}$-PEG_{100}	17.95
$aPCE_{30}$-PEG_{200}	18.69
$iPCE_{30}$-PEG_{100}	17.95
$iPCE_{30}$-PEG_{200}	18.69
$aSPCE_{30}$-PEG_{100}	17.81
$iSPCE_{30}$-PEG_{100}	17.81
PEG_{50}-$aPCE_{30}$-PEG_{50}	17.99
PEG_{50}-$aSPCE_{30}$-PEG_{50}	17.86
PEG_{50}-$iPCE_{30}$-PEG_{50}	17.99

Surface tension measurements

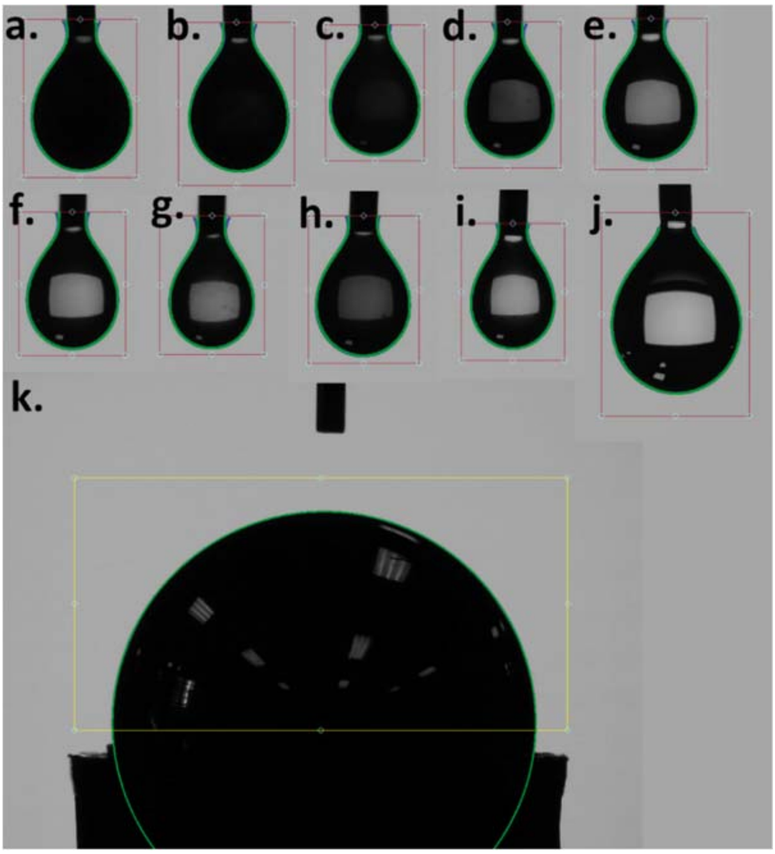

Figure S6. Surface tension measurement of **a)** PEG_{50}-iPCE_{30}-PEG_{50} **b)** PEG_{50}-aSPCE_{30}-PEG_{50} **c)** aPCE_{30}-PEG_{100} **d)** iSPCE_{30}-PEG_{100} **e)** iPCE_{30}-PEG_{200} **f)** aPCE_{30}-PEG_{200} **g)** iPCE_{30}-PEG_{100} **h)** PEG_{50}-aPCE_{30}-PEG_{50} **i)** aSPCE_{30}-PEG_{100} and **j)** water. The green line corresponds to the detected edge of the droplet, the blue line corresponds to the simulated fit used to calculate the surface tension. The difference between the simulated edge and the detected edge is the root mean squared error (RMSE). The instrument was calibrated on **k)** a 10.00 mm sphere.

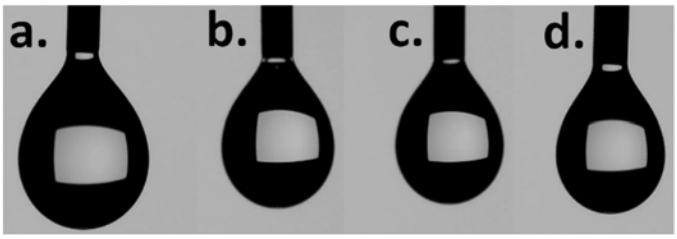

Figure S7. Surface tension measurement of **a)** water **b)** P188 **c)** P407 and **d)** P123.

Table S4. Surface tension values at 20 °C of the different synthesized block copolymer as well as the calculated root mean squared error (RMSE).

Block copolymer	Surface tension (mN/m)	RMSE
Water	71.7	0.6
$aPCE_{30}$-PEG_{100}	32.5	0.3
$aPCE_{30}$-PEG_{200}	36.6	0.4
$iPCE_{30}$-PEG_{100}	30.8	0.6
$iPCE_{30}$-PEG_{200}	37.7	0.4
$aSPCE_{30}$-PEG_{100}	26.7	0.4
$iSPCE_{30}$-PEG_{100}	35.1	0.4
PEG_{50}-$aPCE_{30}$-PEG_{50}	42.0	0.5
PEG_{50}-$aSPCE_{30}$-PEG_{50}	42.7	0.5
PEG_{50}-$iPCE_{30}$-PEG_{50}	46.5	0.5

Emulsion pictures

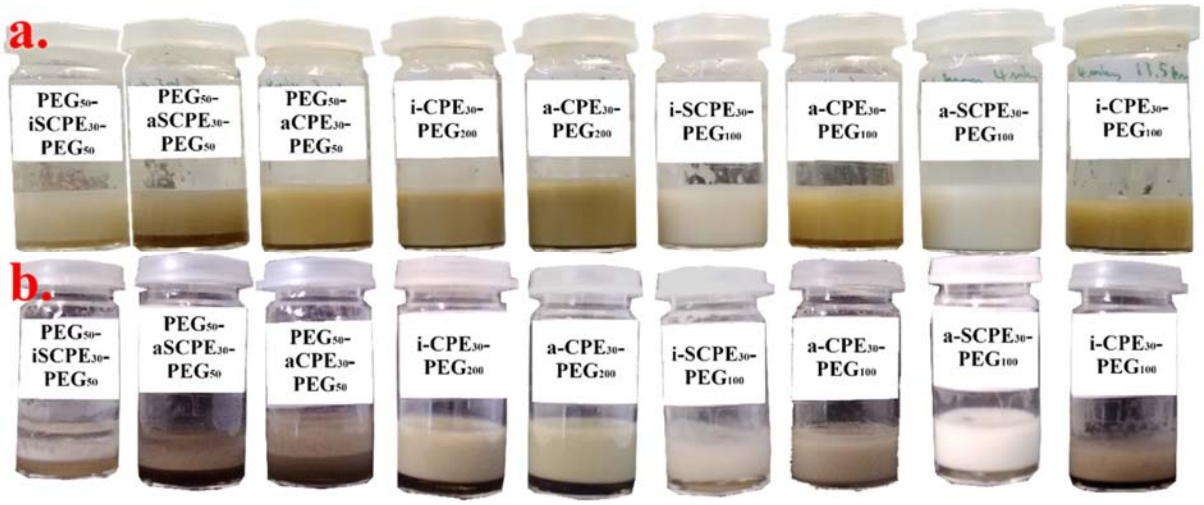

Figure S8. Emulsion pictures of different PEG-PCE block copolymers at 0.5 wt.% concentration and a 65:35 n-dodecane/water ratio after **a)** 1 day and **b)** 4 months.

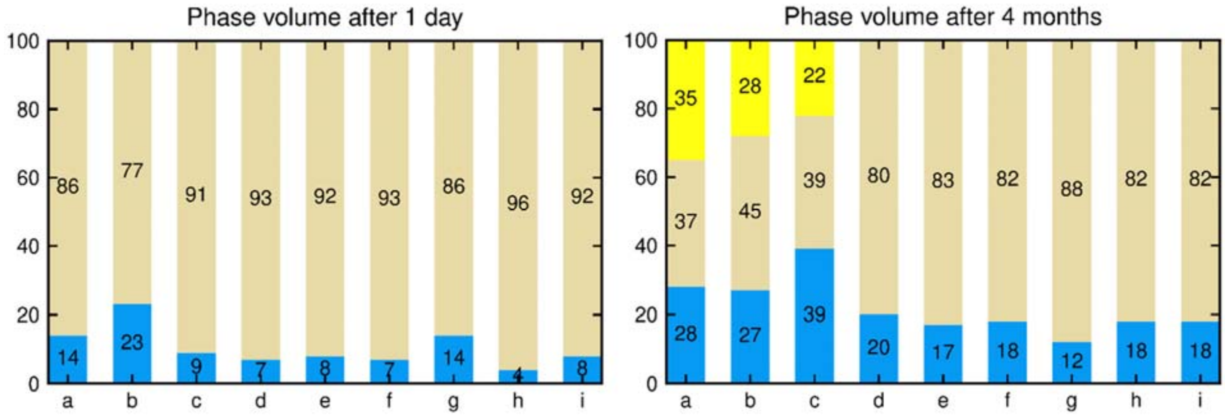

Figure S9. Volume of the oil phase (top, yellow), emulsion phase (middle, beige) and water phase (bottom, blue) of emulsions prepared with PEG-PCE block copolymer at 0.5 wt.% concentration and a 65:35 n-dodecane/water ratio after 1 day and 4 months. **a)** PEG_{50}-i$SPCE_{30}$-PEG_{50}, **b)** PEG_{50}-a$SPCE_{30}$-PEG_{50}, **c)** PEG_{50}-aPCE_{30}-PEG_{50}, **d)** iPCE_{30}-PEG_{200}, **e)** aPCE_{30}-PEG_{200}, **f)** i$SPCE_{30}$-PEG_{100}, **g)** aPCE_{30}-PEG_{100}, **h)** a$SPCE_{30}$-PEG_{100} and **i)** iPCE_{30}-PEG_{100}.

Emulsion microscopy

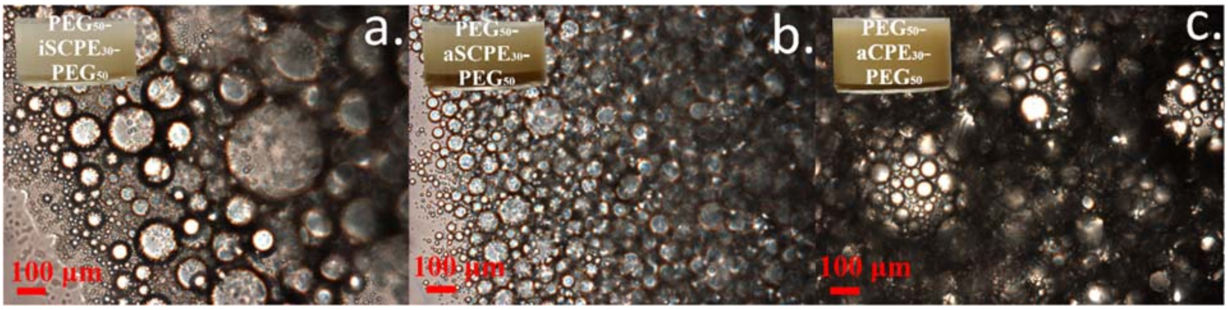

Figure S10. Optical microscopy pictures of **a)** PEG$_{50}$-iSCPE$_{30}$-PEG$_{50}$, **b)** PEG$_{50}$-aSCPE$_{30}$-PEG$_{50}$, **c)** PEG$_{50}$-aCPE$_{30}$-PEG$_{50}$ block copolymer emulsions, with a polymer concentration of 0.5 wt.% and a 65:35 n-dodecane/water ratio (100 µm scale bar).

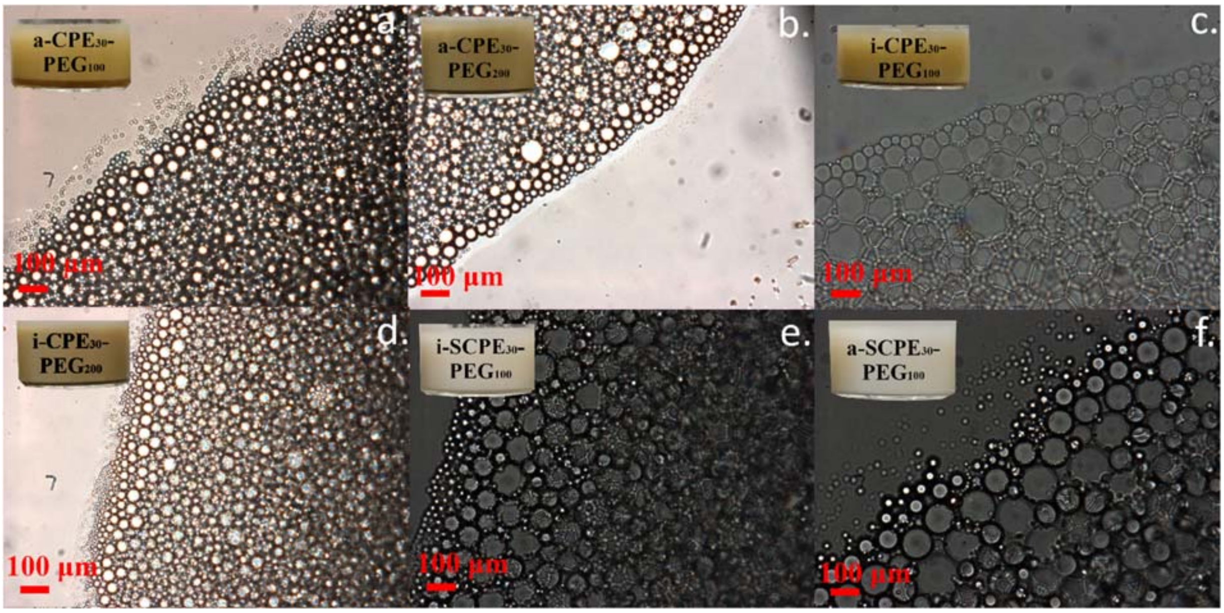

Figure S11. Optical microscopy pictures of **a)** aCPE$_{30}$-PEG$_{100}$, **b)** aCPE$_{30}$-PEG$_{200}$, **c)** iCPE$_{30}$-PEG$_{100}$, **d)** aCPE$_{30}$-PEG$_{200}$, **e)** aSCPE$_{30}$-PEG$_{100}$, **f)** aSCPE$_{30}$-PEG$_{100}$ block copolymer emulsions, with a polymer concentration of 0.5 wt.% and a 65:35 n-dodecane/water ratio (100 µm scale bar).

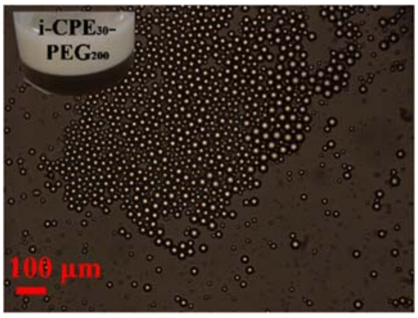

Figure S12. Optical microscopy pictures of iCPE$_{30}$-PEG$_{200}$ block copolymer emulsion, with a polymer concentration of 0.5 wt.% and a 65:35 **toluene**/water ratio (100 µm scale bar).

Emulsion droplet size distribution

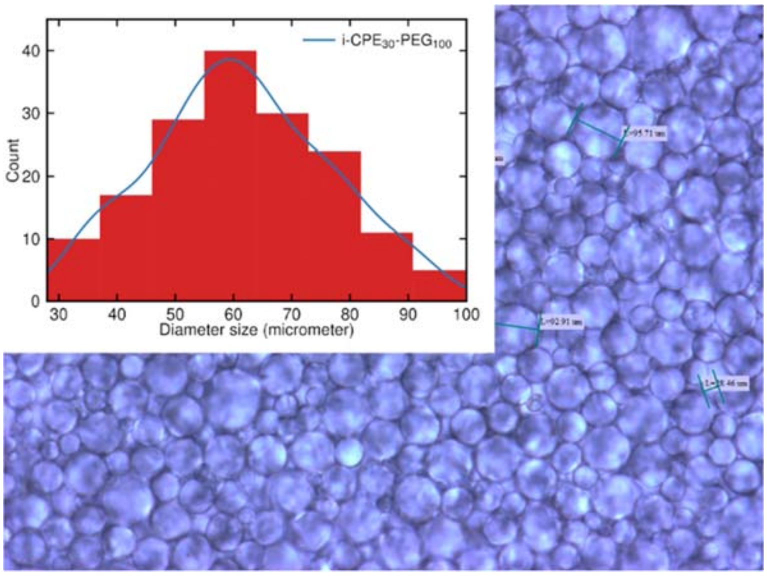

Figure S13. Microscope image of an iPCE$_{30}$-*b*-PEG$_{100}$ emulsion with a polymer concentration of 0.5 wt.% and a 50:50 n-dodecane/water ratio, along with a size distribution of droplet diameters.

Dynamic light scattering characterization of PEG-polycycloether and poloxamers

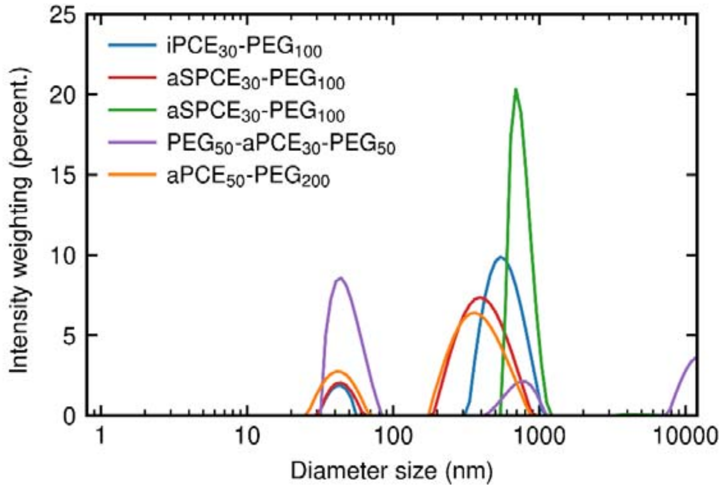

Figure S14. Size of PEG-polycycloether block copolymer self-assemblies in water at a concentration of 5 mg/mL, at 20 °C, after 6 months. Measured by dynamic light scattering, using back-scattering (175°) and intensity weighting.

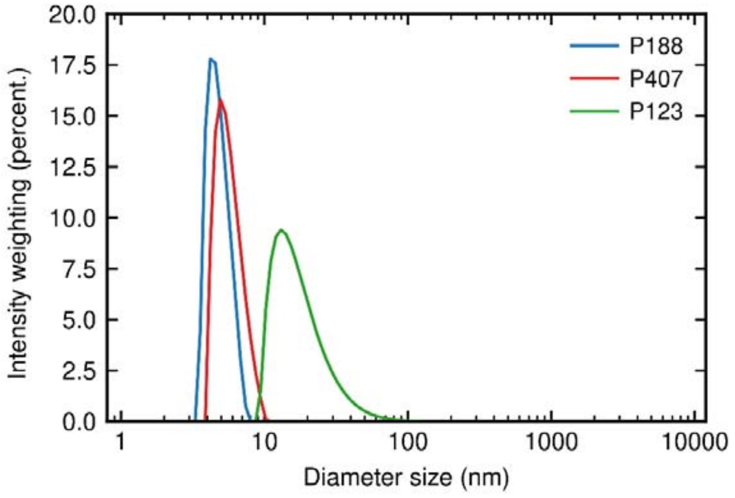

Figure S15. Size of poloxamers P188, P407 and P123 self-assemblies in water, at 20 °C and at a concentration of 5 mg/mL. Measured by dynamic light scattering, using back-scattering (175°) and intensity weighting.

Thermodynamic stability of emulsions formed by PEG-polycycloethers block copolymers

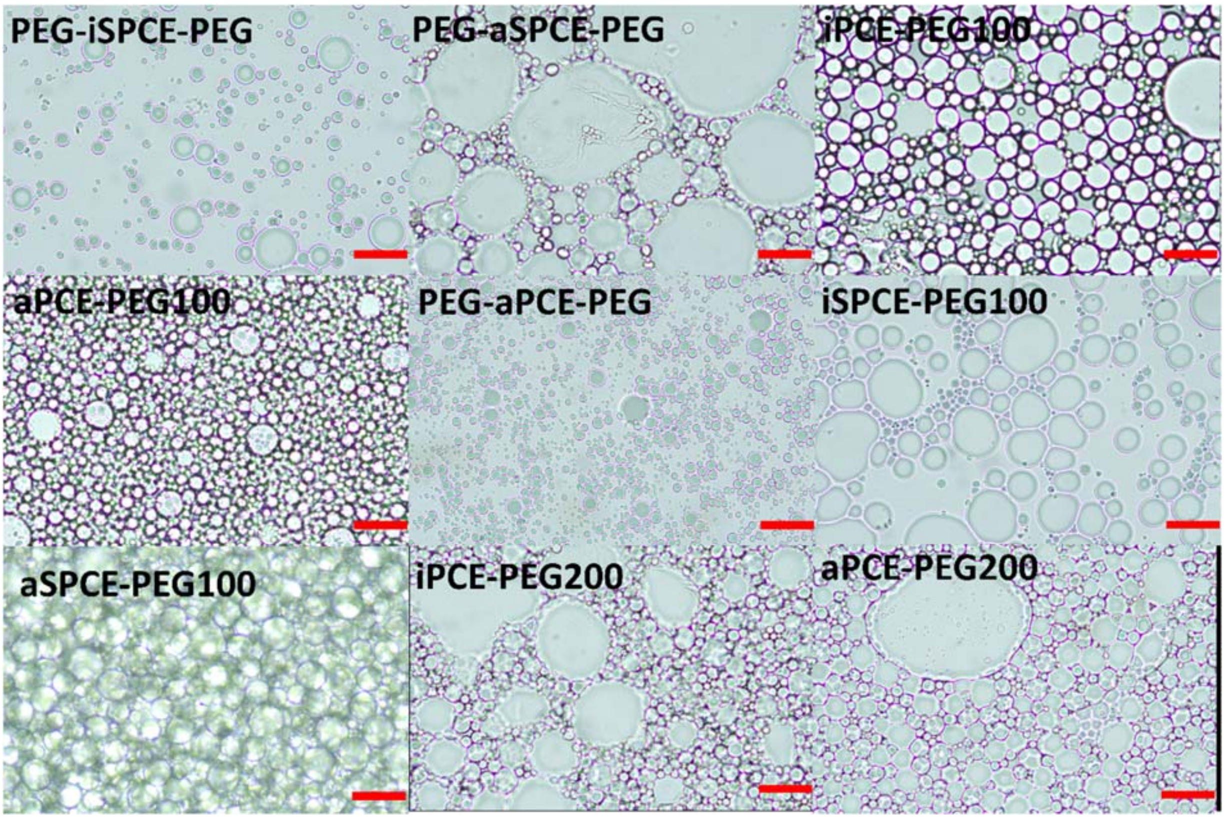

Figure S16. Optical microscopy images of n-dodecane/water 80:20 vol% emulsions formed by PEG-PCE block copolymer after 6 months. Scale bars are 30 µm.

Table S5. Mean diameter size and standard deviation of n-dodecane/water 80:20 vol% emulsion droplets formed by PEG-PCE block copolymers (0.5 wt.% poloxamer concentration) after 6 months.

Polymer	Mean diameter size (μm)	Standard Deviation (μm)
PEG_{50}-i$SPCE_{30}$-PEG_{50}	8.82	5.75
PEG_{50}-a$SPCE_{30}$-PEG_{50}	21.5	21.9
iPCE_{30}-PEG_{100}	14.7	4.93
aPCE_{30}-PEG_{100}	13.0	11.7
PEG_{50}-aPCE_{30}-PEG_{50}	9.52	3.48
i$SPCE_{30}$-PEG_{100}	5.81	2.57
a$SPCE_{30}$-PEG_{100}	14.1	9.50
iPCE_{30}-PEG_{200}	13.3	5.33
aPCE_{30}-PEG_{200}	11.0	3.81

Evaluation of poloxamer P188, P407 and P123 properties

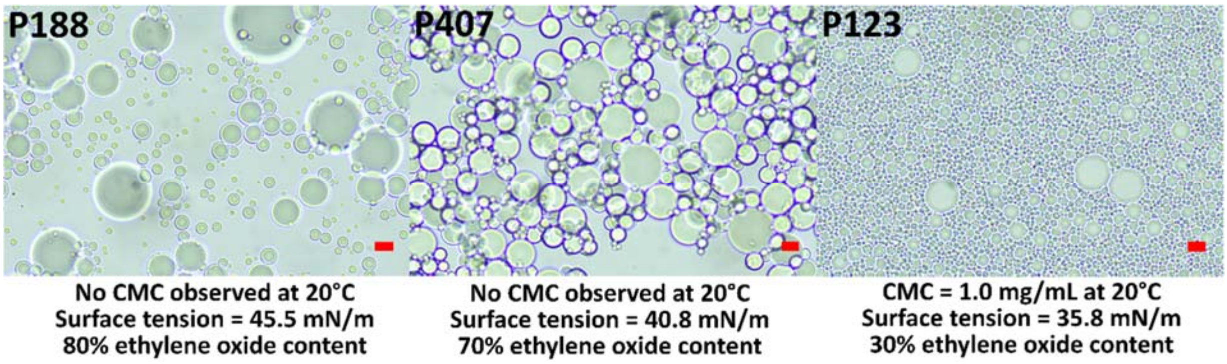

Figure S17. Optical microscopy images of n-dodecane/water 80:20 vol% emulsions formed by poloxamers P188, P407 and P123 after a day. Scale bars are 10 μm. Critical micelle concentration (CMC), surface tension and ethylene oxide content is indicated.

Table S6. Mean diameter size and standard deviation of n-dodecane/water 80:20 vol% emulsion droplets formed by poloxamers P188, P407 and P123 after a day (0.5 wt.% poloxamer concentration).

Poloxamer	Mean diameter size (μm)	Standard deviation (μm)
P188	13.4	6.99
P407	10.4	4.61
P123	3.52	1.29

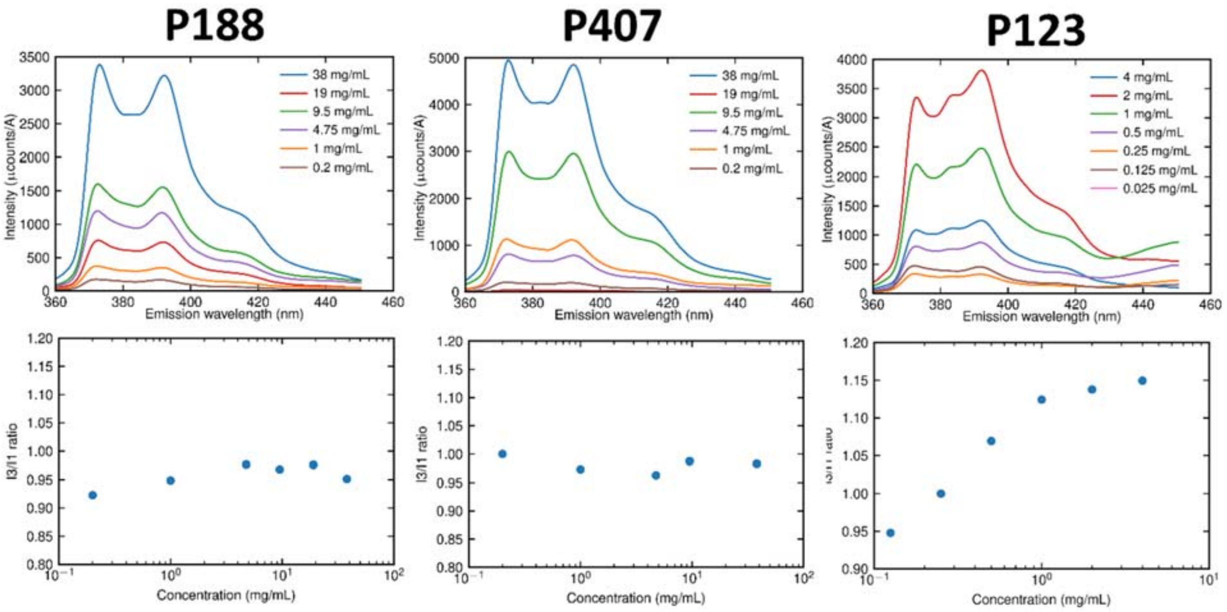

Figure S18. Fluorescence intensity trace for poloxamers P188, P407 and P123 at different concentrations, using pyrene as a fluorescence probe, at 20 °C. CMC determination by plotting I_3/I_1 ratio as a function of concentration. No CMC could be observed for poloxamer P188 and P407.

Determination of the oil-in-water nature of the emulsion

Please refer to the added video file.

Video S1. Video of the droplet test used to determine the nature of the emulsions. $iPCE_{30}$-PEG_{100} was used at a concentration of 0.5 wt% with a 50:50 n-dodecane/water ratio. Rhodamine B was used as a dye.
